# Conventional Antimicrobial and Medicinal Plants from a Traditional Medicine Market in South Africa: An Interactive Antimicrobial and Toxicity Study

**DOI:** 10.3390/antibiotics14050512

**Published:** 2025-05-15

**Authors:** Zelna Booth, Sabiha Essack, Sandy van Vuuren

**Affiliations:** 1 Department of Pharmacy and Pharmacology, Faculty of Health Sciences, School of Therapeutic Sciences, University of the Witwatersrand, Johannesburg 2000, South Africa; sandy.vanvuuren@wits.ac.za; 2 Antimicrobial Research Unit, University of KwaZulu-Natal, Durban 4000, South Africa; essacks@ukzn.ac.za

**Keywords:** antibiotics, antifungals, traditional medicinal plants, antimicrobial combinations, interactions, synergistic, antagonistic, toxicity

## Abstract

Background: The World Health Organization (WHO) has proposed the use of integrative medicine to achieve extended healthcare coverage in developing countries facing high morbidity. Traditional remedies are frequently employed to prevent and treat infections among South Africans; however, the ways in which they interact with conventional antimicrobials are largely unknown. Therefore, this study aimed to explore the interactions between commonly traded medicinal plants at a traditional medicine market in KwaZulu-Natal (KZN), South Africa, and conventional antibiotics and antifungals. Methods: To determine the interactive antimicrobial profiles for plant/conventional antimicrobial combinations, minimum inhibitory concentration (MIC) assays were performed against ESKAPE pathogens and the yeasts *Candida albicans* and *Candida glabrata*. Calculated fractional inhibitory concentration (ΣFIC) values were used to identify synergism or antagonism, with synergistic interactions further tested in vitro for toxicity. Results: A total of 952 combinations were tested, of which 5.8% and 54.6% of the plant/antibiotic combinations were synergistic and antagonistic, respectively; additionally, 1.7% and 58.6% of the plant/antifungal combinations showed synergism or antagonism, respectively. The most toxic plant/antibiotic combination was *Artemisia afra* with doxycycline (71.1% mortality). The most toxic plant/antifungal combination was *Acorus calamus* with fluconazole (78.8% mortality). Conclusions: When medicinal plants acquired from a traditional medicine market in South Africa are used in combination with conventional antibiotics and antifungals, more than half of the combinations exhibit antagonism, which is concerning.

## 1. Introduction

An estimated 60–80% of South Africans make use of traditional medicines sourced from various traditional medicine markets [1,2,3]. With the country’s diverse array of indigenous plants, exposure to a wide range of traditional medicinal plants is highly prevalent among the population, particularly for the treatment of infectious conditions [4]. Given the widespread use of medicinal plants in South Africa and the substantial burden of infections [5], there is a significant likelihood of combined traditional and conventional antimicrobial use.

Consequently, it is crucial to investigate the interactions of these combinations. Synergistic antimicrobial interactions could prove beneficial as alternatives to addressing resistant infections. The potentiation of antimicrobial efficacy could be harnessed without having to increase dosing, which subsequently reduces toxicity. Of concern, however, is the potential for antagonism. Specifically, the findings from a recent study on antimicrobial interactions between traditional medicinal plants purchased from an informal Johannesburg (JHB) traditional medicine market (Faraday market) and conventional antimicrobials, revealing that most combinations were antagonistic [6].

This study aimed to investigate whether this pattern of antagonism extends to traditional medicinal plants with varied geographic distributions in South Africa. Specifically, we evaluated the in vitro antimicrobial interactions and toxicity profiles of commonly used traditional medicinal plants from KwaZulu-Natal (KZN), traded at the Warwick market, when used in combination with conventional antimicrobials against selected bacterial and fungal species.

## 2. Results

### 2.1. Individual Antimicrobial Activity of Plants

This study focused specifically on combinations, but, to evaluate interactions, baseline minimum inhibitory concentration (MIC) values for individual samples needed to be established. All antimicrobials were found to be aligned with the EUCAST [7]-CLSI [8] breakpoint ranges (Supplementary Tables S1 and S2), and the individual plant extract activities against the ESKAPE pathogens (*Enterococcus faecium* ATCC 27270, *Staphylococcus aureus* ATCC 25923; *Klebsiella pneumoniae* ATCC 13883, *Acinetobacter baumannii* ATCC 19606, *Pseudomonas aeruginosa* ATCC 27853, and *Enterobacter cloacae* NCTC 13406; and two yeasts (*Candida albicans* ATCC 10231 and *Candida glabrata* ATCC 90030) are summarized in Supplementary Tables S1 and S2, respectively. An evaluation of the individual plant samples for antimicrobial activity established that *Elaeodendron transvaalense* (organic extract) was the most active amongst the plant samples when tested against *A. baumannii*, with an MIC of 0.4 mg/mL (Supplementary Table S1).

### 2.2. Plant Extracts in Combination with Commercial Antimicrobials

**Figure 1:**
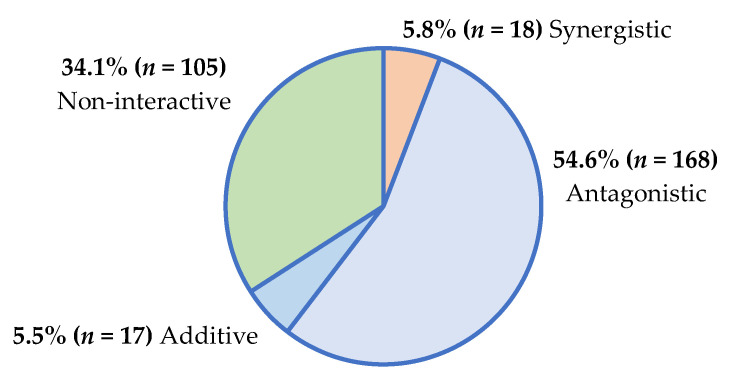
Interactive antimicrobial profiles of plant/antibiotic combinations (*n* = 308) against ESKAPE pathogens.

A total of 952 combinations of plant (aqueous and organic) extracts with conventional antimicrobials were tested against the six ESKAPE pathogens and the two yeasts (Supplementary Figures S1–S19). When the extracts of the 17 plants (50 µL of 32 mg/mL extract) were combined with antimicrobials (50 µL of 0.01 mg/mL antibiotic, or 50 µL of 0.1 mg/mL antifungal) and tested against their respective pathogens, many combinations lacked definitive interactive ΣFIC classifications, since the MIC endpoints could not be reached (i.e., MIC > 4 mg/mL for plant extracts or MIC >1.25 µg/mL for antibiotics and >12.5 µg/mL for antifungal agents). The interactive summary of the 308 plant/antibiotic combinations with definitive interactive values, determined using the sum of the fractional inhibitory concentration (ΣFIC) calculation (Supplementary Figures S1–S17), is presented in Figure 1.

**Figure 2:**
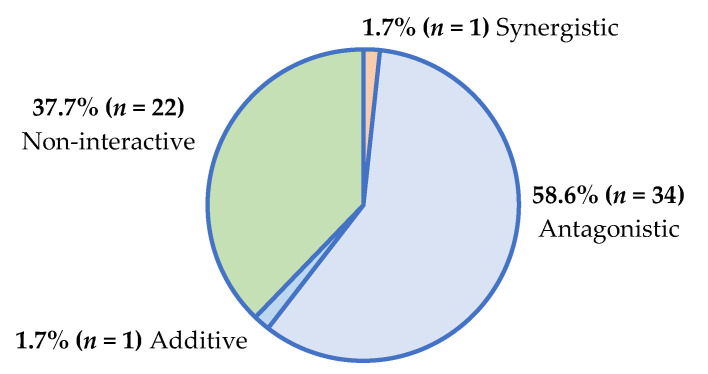
Interactive antimicrobial profiles of plant/antifungal combinations (*n* = 58) against yeasts.

Furthermore, the interactive classification overview of the 58 plant/antifungal combinations with definitive ΣFIC values (Supplementary Figures S18 and S19) is presented in Figure 2.

Most plant/antimicrobial combinations demonstrated antagonism, which is concerning. Regarding the treatment of infectious conditions, antagonistic interactions pose a threat to the efficacy of antimicrobial therapies. Reduced antimicrobial potency further promotes the development of antimicrobial resistance among pathogens, rendering the existing antimicrobial agents ineffective [9].

Some interactions were synergistic (5.8% of plant/antibiotic combinations; 1.7% of plant/antifungal combinations), and these interactions could lead to more effective therapeutic outcomes that could prevent antimicrobial resistance. As additive and non-interactive profiles have little therapeutic impact, the combinations demonstrating antagonism (reduced antimicrobial efficacy) and synergy (enhanced antimicrobial efficacy) are only further described to emphasize their potential therapeutic impact on antimicrobial outcomes.

#### 2.2.1. Plant Combinations with Amoxicillin

Among the plant extract combinations with amoxicillin that have a definitive ΣFIC (*n* = 182), no synergistic interactions were identified, with 26.4% of the combinations being antagonistic (*n* = 48). Plant combinations with amoxicillin were most antagonistic against *A. baumannii*, where 16 of the 17 tested plants were antagonistic in combination (Table 1). The most prominent antagonistic interaction was between *S. serratuloides* (aqueous extract) and amoxicillin, yielding a ΣFIC of 135.49 against *K. pneumoniae*. No synergistic interactions were exhibited for further reporting.

#### 2.2.2. Plant Combinations with Azithromycin

Azithromycin is not typically effective against many ESKAPE pathogens; however, it is a broad-spectrum antibiotic that is frequently prescribed, and unnecessarily overused, in conventional healthcare settings [10]. Among the plant extract combinations with azithromycin that had a definitive ΣFIC (*n* = 77), 11.7% were synergistic (*n* = 9) and 54.6% were antagonistic (*n* = 42) (Table 2). The greatest antagonism was noted for plant/azithromycin combinations when tested against *P. aeruginosa*, where 16 of the 17 combinations were antagonistic. Synergism was observed only against the Gram-positive pathogens, *S. aureus* and *E. cloacae*, where *J. zeyherii* (organic extract) with azithromycin against *S. aureus* had the greatest synergistic effect (ΣFIC of 0.22).

#### 2.2.3. Plant Combinations with Ciprofloxacin

Among the plant extract combinations with ciprofloxacin with a definitive ΣFIC (*n* = 77), 2.6% were synergistic (*n* = 2) and 64.9% were antagonistic (*n* = 50) (Table 3). The greatest antagonism was evident when combinations were tested against *S. aureus* and *A. baumannii*, where 14 and 15, respectively, of the 17 plant sample combinations were antagonistic.

Only two synergistic interactions were noted for the plant/ciprofloxacin combinations; however, overall, the most synergistic interaction for this study was identified between *Strychnos henningsii* and ciprofloxacin against *P. aeruginosa* (ΣFIC of 0.07). Conversely, the most notable antagonistic interaction was found for *S. serratuloides*/ciprofloxacin against *A. baumannii* (ΣFIC of 139.87).

#### 2.2.4. Plant Combinations with Doxycycline

Among the plant/doxycycline combinations with definitive ΣFIC values (*n* = 91), 7.7% were synergistic (*n* = 7) and 30.8% were antagonistic (*n* = 28) (Table 4). The most notable synergism was between *R. melanophloeos*/doxycycline against *E. cloacae* (ΣFIC of 0.30). Four plant organic extracts (*H. africana*, *J. zeyheri*, *S. hyacinthoides*, and *S. serratuloides*) with doxycycline against *A. baumannii*, were most antagonistic (ΣFIC of 24.50).

#### 2.2.5. Plant Combinations with Nystatin

Among the plant extract combinations with nystatin that had a definitive ΣFIC value (*n* = 25), no synergy was observed, and a considerable 60.0% antagonism was noted against the yeasts *C. albicans* and *C. glabrata* (Table 5). The combination of *H. africana* (organic extract) and nystatin against *C. albicans* was the most antagonistic (ΣFIC of 19.67) among the antifungal combinations. A greater frequency of antagonism was noted with the organic extracts (*C. albicans* where *n* = 8) than with the aqueous extracts (*C. albicans* where *n* = 1) for the plant/nystatin combinations. However, both the organic and aqueous extracts in combination with nystatin had the same frequency of antagonistic effects on *C. glabrata* (*n* = 3). No synergy was observed for conventional antifungal agents.

#### 2.2.6. Plant Combinations with Fluconazole

For the plant/fluconazole combinations with a definitive ΣFIC value (*n* = 33) against *C. albicans* and *C. glabrata*, only 3.0% were synergistic (*n* = 1), and 57.6% were antagonistic (*n* = 19) (Table 6). The only synergistic interaction was identified for the combination of *A. calamus* (organic extract) and fluconazole, with *C. albicans*, yielding a ΣFIC value of 0.34. The most antagonistic combination was exhibited when *R. melanophloeos* (organic extract) was combined with fluconazole and *R. tridentata* (organic extract) with fluconazole, both of which were tested against *C. glabrata*, yielding a ΣFIC of 16.08.

### 2.3. Toxicity Studies

The relevant samples were tested for toxicity individually via the brine shrimp lethality assay (BSLA) (Supplementary Table S3). To determine whether synergism was reflective of toxicity or the true increased antimicrobial potency of a combination, only plant samples that demonstrated synergistic interactions with conventional antimicrobials were tested for toxicity (Table 7).

Among the individual plant extracts studied, only the *A. calamus* and *E. transvaalense* organic extracts were found to be toxic, with average mortality rates of 100.0% and 55.8%, respectively (Supplementary Table S3).

Among the 17 synergistic combinations evaluated for toxicity (Table 7), only four (*A. afra* organic extract with doxycycline 71.1%; *R. melanophloeos* organic extract with doxycycline 51.3%; *G. perpensa* aqueous extract with doxycycline 57.5%; and *A. calamus* organic extract with fluconazole 78.8%) were toxic after only 48 h of exposure.

The independent plant samples that were toxic had reduced mortality rates when combined, except for the *A. calamus* (organic extract)/fluconazole combination, where considerable toxicity was still evident (78.8% average mortality after 48 h). This combination was also the only combination with an antifungal agent that was found to have synergistic antimicrobial effects. When the *A. calamus* (organic extract)/azithromycin combination was tested, the toxicity was reduced to 27.5% for the combination.

## 3. Discussion

Combinations of various antimicrobials with plant extracts demonstrating pertinent synergistic and antagonistic profiles were evaluated in this study. They may present enhanced or reduced antimicrobial activity in terms of the therapeutic impact.

Amoxicillin belongs to the penicillin family and is commonly used for the treatment of bacterial infections of the respiratory tract, skin, and oral cavity [10,11,12,13]. *Staphylococcus aureus* poses a serious health threat, as the pathogen has intrinsic virulence, the ability to cause a diverse array of life-threatening infections, and the capacity to adapt to different environmental conditions [14,15]. Furthermore, a literature review was conducted [9] in which a surge in the resistance of *S. aureus* toward amoxicillin was frequently reported in the literature. The identification of new, more effective antimicrobial options is therefore paramount, and synergistic plant: antimicrobial combinations, could serve as alternative avenues for investigation. Conversely, antagonistic plant/antimicrobial combinations could further exacerbate the health threat posed by resistant Staphylococcal infections.

*Lippia javanica, A. afra, R. melanophloeos, R. caffra* and *S. serratuloides*, which are traditionally used for the treatment of respiratory tract infections [16,17,18] (Supplementary Table S4), all exhibited antagonism in combination with amoxicillin, particularly against three prominent causative pathogens of respiratory tract infections (*A. baumannii, S. aureus* and *K. pneumoniae*).

Antibacterial activity of *A. afra* (organic extract) was investigated [19] and it was reported that *A. afra* was highly active against *S. aureus*, and active even against methicillin-resistant *S. aureus*. With amoxicillin commonly employed to treat respiratory tract infections in conventional healthcare settings [10,11,12,13], it is likely that the combination of amoxicillin with medicinal plants is used traditionally for similar respiratory complaints. Considering the antagonism identified in the current study, a combination of this nature should not be used.

Amoxicillin is also frequently used for the treatment of skin infections in conventional health settings [10,11,12,13,20]. *Berchemia discolor, G. perpensa*, *H. africana*, *J. zeyheri*, *L. javanica*, *R. caffra*, *S. serratuloides*, *S. henningsii* and *W. salutaris* are all traditionally used for the treatment of skin infections, particularly in wound treatment [16,17,18] (Supplementary Table S4). Hence, with the prevalence of antagonism between amoxicillin and these medicinal plants is cause for concern, particularly against the pathogens *S. aureus*, *P. aeruginosa*, *A. baumannii, E. cloacae* and *E. faecium*, which are known for their potential to infect wounds [10]. Although *S. aureus* is a natural skin commensal, when the skin is perforated by injury, the bacteria can become pathogenic and result in soft skin tissue infections [20]. *Staphylococcus aureus* has become resistant to many ß-lactam antibiotics, including amoxicillin [15,19] and it emphasizes that the rapid development of multidrug-resistant *S. aureus* has resulted in difficulties in achieving effective treatment [20]. Therefore, the antagonism noted in the present study warrants concern in relation to the efficacy of amoxicillin in the treatment of infections caused by *S. aureus*. 

Azithromycin is a macrolide antibiotic used to treat respiratory tract infections (pneumonia and sinusitis), in addition to gastro-intestinal and skin infections [10,11,12,13]. Azithromycin, exhibits known activity against both *P. aeruginosa* and *S. aureus* [10,11,12,13]. Furthermore, *S. serratuloides* is traditionally used for respiratory tract infections and was found to be synergistic with azithromycin against *S. aureus* (ΣFIC of 0.50). This combination is promising for treating *S. aureus* sinusitis in the future, particularly since this combination was further found to be non-toxic (Table 7).

Conversely, antagonism between 16 of the 17 plant samples tested with azithromycin against *P. aeruginosa* is worrisome, since azithromycin is clinically employed for the treatment of *P. aeruginosa* infections [10,11,12,13].

Azithromycin is further used to treat various gastrointestinal complaints and associated infections, such as traveler’s diarrhoea [10,11,12,13]. Both *C. laureola* and *E. transvaalense* are used traditionally for gastro-intestinal infections [16,17,18] (Supplementary Table S4). Given that *S. aureus* is causative of food poisoning, and that azithromycin is a potential conventional treatment [10,11,12,13], these synergistic interactions between traditional and conventional medicines should be investigated further. Generally, aqueous extracts exhibit poor antimicrobial activity. Interestingly, however, in this study, aqueous extracts of *G. perpensa* (ΣFIC of 0.36) and *B. discolor* (ΣFIC of 0.49) combined with azithromycin and tested against *E. cloacae* were synergistic, providing further impetus for further exploration. These synergistic combinations offer an even more favorable approach for treating resistant skin infections, since combinations were also found to be non-toxic in the present study.

A further potential combination is *J. zeyheri* with azithromycin for the treatment of skin infections [16,17,18] (Supplementary Table S4), since synergism (ΣFIC of 0.22) against *S. aureus* was observed, along with a lack of toxicity in the BSLA (Table 7). Azithromycin is often prescribed in conventional healthcare settings [10,11,12,13], and *J. zeyheri* [16,17,18] (Supplementary Table S4) is used traditionally for the treatment of skin infections. *Staphylococcus aureus* commonly causes skin infections, with ailments such as boils, abscesses, cellulitis, atopic dermatitis, impetigo, folliculitis, and secondary wound infections [10]. Hence, this synergistic interaction should be explored further to determine its clinical relevance, particularly since *S. aureus* has the potential to rapidly develop resistance toward conventional antibiotic treatments, often resulting in resistance to multiple antibiotics (multidrug-resistant *S. aureus*) [20].

Ciprofloxacin is a broad-spectrum antibiotic from a group of antibiotics classified as fluoroquinolones [10,11,12,13]. Ciprofloxacin is used in the clinical setting to treat uncomplicated urinary tract infections and respiratory tract infections, such as pneumonia, as well as skin, bone, eye and ear infections [10,11,12,13].

*Strychnos henningsii* is traditionally used for the treatment of gynecological, respiratory, oral cavity, skin, and gastro-intestinal infections [16,17,18] (Supplementary Table S4). *Pseudomonas aeruginosa* is a Gram-negative pathogen that causes bloodstream and respiratory tract infections, as well as infections of other parts of the body following surgery (nosocomial infections) [10,11,12,13]. The organic extract of *S. henningsii* with ciprofloxacin demonstrated the most noteworthy synergistic interaction when tested against *P. aeruginosa* (ΣFIC of 0.07). Antibiotic resistance is prominent in Pseudomonal infections [10,11,12,13], so the identified synergistic interaction could be explored as a mechanism to overcome this resistance, particularly since the combination is non-toxic (Table 7) and ciprofloxacin is an antibiotic that is used extensively in treating *P. aeruginosa* infections [10,11,12,13].

Doxycycline is a tetracycline antibiotic [11]. It is used for the treatment of respiratory, skin and dental infections [10,11,12,13]. Doxycycline is often employed in conventional treatments for skin infections [10,11,12,13]. With *B. discolor* being traditionally used for skin infections [16,17,18] (Supplementary Table S4), there is an alignment between traditional and conventional treatments, which could be of clinical relevance.

Nystatin is an antimicrobial used to treat fungal or yeast infections of the skin and oral cavity [10,11,12,13]. *Candida* spp. are well known for causing topical yeast infections, particularly in immunocompromised patients [10]. *Hydnora africana* is used traditionally to treat skin infections [16,17,18] (Supplementary Table S4). Hence, the antagonism documented in this study is a cause for concern in the treatment efficacy of conventional nystatin. For skin infections caused by both *C. albicans* and *C. glabrata*, there is concern should a patient make use of *H. africana* simultaneously with nystatin.

Fluconazole is an antifungal agent used to treat infections of the skin, genitalia, and oral cavity [10,11,12,13]. It is also employed for the treatment of systemic Candidal infections [10,11,12,13]. The only synergistic antifungal interaction was with *A. calamus* and fluconazole against *C. albicans*. This combination is even more promising considering the lack of toxicity in the BSLA (Table 7). *Acorus calamus* has been studied for its antimicrobial, anthelmintic, antidiarrheal, antioxidant, anti-ulcer, and analgesic activities, among other properties [21,22]. Moderate antifungal activity of *A. calamus* extracts against *C. albicans* has been reported [22,23,24]. However, there are few studies on the combination of *A. calamus* with conventional antifungals. A study that explored their combined use was conducted, where *A. calamus* compounds were tested against *C. albicans* in combination with the antifungal’s amphotericin B, clotrimazole and fluconazole and synergy was predominantly observed [23].

A similar study [6] investigated the interactions between conventional antimicrobials (amoxicillin, azithromycin, ciprofloxacin, doxycycline, nystatin and fluconazole) and traditional medicinal plants purchased from a Johannesburg (JHB) traditional medicine market (Faraday market) in Gauteng, South Africa [6]. A total of 816 plant/antibiotic combinations were tested against the ESKAPE pathogens, and synergistic (6.6%) and antagonistic (47.5%) effects were observed. Interactive profiles documented for JHB plant/antibiotic combinations [6] are similar to those obtained in the current study on KZN plant/antibiotic combinations (5.8% synergy; 54.6% antagonism). Furthermore, the greatest synergy was observed in both studies when JHB or KZN plants were combined with azithromycin and tested against the ESKAPE pathogens. Five common medicinal plants were tested in both the previous JHB study and the current KZN study, including *A. calamus*, *H. africana*, *R. caffra*, *S. serratuloides* and *W. salutaris*. Among these, the only shared interactive outcome was evident with *H. africana*/azithromycin combinations against *S. aureus*, where synergy was observed despite *H. africana* being purchased from alternate sources (JHB ΣFIC of 0.5; KZN ΣFIC of 0.5).

The pathogens with the greatest antagonism were *P. aeruginosa* and *A. baumannii*. This is concerning, since these two pathogens pose challenges to existing antimicrobial therapies because of resistance mechanisms [10,11,12,13]. Infections caused by *A. baumannii* are increasing, and these infections are resistant to conventional antibiotics because of their ability to develop biofilms [24].

Interactive findings for the plant/antifungal combinations used in the present study were similar to those previously studied [6], where the interactions were predominantly antagonistic (JHB 61.9%; KZN 58.6%). However, the synergy in the KZN study for plants with antifungal combinations (1.7%) was greater than that previously reported [6] for JHB plant combinations with antifungal agents, where no synergy was observed. Limited evidence of synergistic interactions with the antifungals that are clinically used against pathogenic fungi has been highlighted [25]. The findings from this study, therefore, offer valuable insights into the interactive nature of antifungals with medicinal plants, which are rarely studied or documented in literature.

The strong antagonism evident in the current study requires further investigation as a potential cause of rapidly increasing resistance patterns toward conventional antifungal treatments. This is a serious public health threat, particularly in low- to middle-income countries, such as South Africa, where immunocompromising factors exist [25]. Furthermore, reports [26,27,28] have shown that candidiasis is still strongly associated with HIV infection. The antagonism evident in the present study between the plant/antifungal combinations against two common *Candida* species could be a compounding factor in antifungal resistance development, increasing the health threat posed by *Candida* opportunistic infections among HIV-infected patients.

When both South African studies were compared, a similar number of plant species were antagonistic with nystatin (*n* = 11) and fluconazole (*n* = 13) against both *C. albicans* and *C. glabrata*, indicating that the pattern of antagonism remained constant. This pattern should be further evaluated against other *Candida* strains to substantiate the commonality of the antagonism, although *C. albicans* remains the most common causative pathogen for fungal infections [29]. Non-albicans fungal infections are increasing and include *C. tropicalis*, *C. glabrata, C. parapsilosis*, and *C. krusei* [30,31,32]. Consequently, identifying antifungal therapies with broad-spectrum activity is vital.

The limited range of effective conventional antifungal agents used to treat fungal infections exacerbates the health threat they pose, even more so because antimicrobial resistance has now extended its reach [29,30,31,32]. The findings of the present study underscore that combination therapy is not a feasible option for identifying alternative, more effective treatment options for Candidal infections. 

## 4. Materials and Methods

### 4.1. Plant Material Preparation

The plant samples (*n* = 24) were purchased from commercial traditional medicine traders at the Warwick traditional medicine market in Durban, KZN (South Africa), in February 2022. Appropriate assistance was garnered to facilitate effective communication with traders in the isiZulu local language. The purchases were made in accordance with those products most frequently sold to individuals presenting symptoms of an infectious disease. Services were garnered from a qualified botanist, with extensive knowledge of the plant material traded at the Warwick informal market and who was in good standing with the traders for communication purposes. Botanical identification of the plant material purchased for analysis was based on the physical properties (appearance and sensory) of the plant material. Seven plant samples that could not be botanically verified were removed from the study, and seventeen medicinal plant samples (Supplementary Table S4) remained for in vitro combination antimicrobial testing.

The raw plant materials (leaves/stems/roots/bulbs) were dried and macerated to prepare both aqueous and organic extracts. The organic extracts were prepared by ensuring plant material immersion (20 g of plant material per 250 mL of solvent) in a 1:1 mixture of dichloromethane and methanol for 24 h at 37 °C. Aqueous extracts were obtained by soaking the plant material in sterile water under the same conditions. The resulting filtrates were either evaporated, through exposure to fume hood conditions, to remove solvents (organic extracts) or frozen at −70 °C and lyophilized via a Virtus freeze dryer (aqueous extracts). To ensure sterility, aqueous extracts were exposed to ultraviolet light. Both types of extracts were prepared at a concentration of 32 mg/mL for antimicrobial testing; sterile water was used for the aqueous extracts, and acetone was used for the organic extracts.

### 4.2. Interactive Antimicrobial Analysis

The ESKAPE (*Enterococcus faecium* ATCC 27270, *Staphylococcus aureus* ATCC 25923; *Klebsiella pneumoniae* ATCC 13883, *Acinetobacter baumannii* ATCC 19606, *Pseudomonas aeruginosa* ATCC 27853, and *Enterobacter cloacae* NCTC 13406) pathogens and two yeasts (*Candida albicans* ATCC 10231 and *Candida glabrata* ATCC 90030) (American Type Culture Collection purchased from Davies Diagnostics (Randburg, South Africa)) were cultured in optimal growth media, Tryptone Soya broth (TSB) (Sigma–Aldrich, Johannesburg, South Africa), at 37 °C for 24 h and 48 h at 37 °C for the bacteria and yeasts, respectively. Pathogens were selected due to their ability to cause prominently occurring infectious diseases that are considered by the World Health Organization (WHO) to be “critical or high priority” on their global pathogen list because of their significant impact on morbidity and mortality rates [33].

The conventional antimicrobials, amoxicillin (purity ≥ 95%), azithromycin (purity ≥ 98%), ciprofloxacin (purity ≥ 98%), doxycycline (purity ≥ 93.5%), nystatin (purity ≥ 98%) and fluconazole (purity ≥ 98%) (Sigma–Aldrich, Johannesburg, South Africa), which are regularly used in the public healthcare sector against the selected pathogens, were prepared accordingly for analysis [12].

The antimicrobial activities of the plant samples (100 μL) and conventional antimicrobials (100 μL) were determined, after which the plant/antimicrobial combinations (50 μL of each agent of the combination) were assessed via the minimum inhibitory concentration (MIC) assay and CLSI [8]-approved transfer techniques. The positive control of ciprofloxacin 0.01 mg/mL or nystatin 0.1 mg/mL and negative (32 mg/mL of acetone in water) controls, due to spectrum of activity, along with culture control (TSB), were included. After the serial doubling dilution method was completed, the 0.5 McFarlands turbidity standard was added, and the plates were tightly sealed and incubated accordingly (bacteria were incubated at 37 °C for 24 h and yeasts were incubated at 37 °C for 48 h). The *p*-iodonitrotetrazolium violet (0.4 mg/mL) (Sigma–Aldrich, Johannesburg, South Africa) indicator was added to all the wells at 40 μL and left until the culture control turned purple/pink in the presence of microbial growth, with MIC readings taken thereafter at the lowest concentration to inhibit microbial growth (the first well had no pink-purple coloring from the indicator) [34]. All individual samples and combinations were tested in duplicate or triplicate when needed.

### 4.3. Fractional Inhibitory Concentration Index

The sum of the fractional inhibitory concentration index (ΣFIC) was calculated according to [31], using the equation ΣFIC = FIC ^(i)^ + FIC ^(ii)^, where (a) represents the plant sample and (b) the conventional antimicrobial sample:

FIC ^(i)^ = MIC (a) in combination with (b)

MIC (a) independently

FIC ^(ii)^ = MIC (b) in combination with (a)

MIC (b) independently

The interactions with definitive ΣFIC values (MIC endpoints reached) were classified as synergistic (ΣFIC ≤ 0.5), additive (ΣFIC > 0.5–1.0), noninteractive (ΣFIC > 1.0 and ≤4.0) or antagonistic (ΣFIC > 4.0) [34]. Since combinations demonstrating synergy or antagonism are highly relevant to antimicrobial treatment outcomes, these two classifications were the focus of this study [6].

### 4.4. Toxicity Studies

Only synergistic antimicrobial combinations were tested for toxicity to determine whether the observed synergism was due to enhanced antimicrobial activity or was simply reflective of toxicity. Tropic Marine^®^ Sea Salt (32 g) was dissolved in 500 mL of distilled water to prepare artificial saltwater for the addition of dried brine shrimp (*Artemia franciscana*) larvae (0.5 g) (Ocean Nutrition™, Cape Town, South Africa). The incubation conditions included constant light (LED bulb, 220–240 V), rotary pump aeration (Kiho), and maintenance at 25 °C for 24–48 h [35].

After hatching, 400 μL of saltwater containing live brine shrimp was added to each well of a 48-well sterile microtiter plate. Subsequently, 400 μL of the sample (individual plant extracts, antimicrobials, or their combinations) was prepared at 2 mg/mL in sterile water (aqueous extracts) or 2% *v/v* dimethyl sulfoxide (DMSO) (organic extracts) and added to the wells in triplicate, resulting in a final test concentration of 1 mg/mL [35,36]. The negative control consisted of 32 g/L saltwater, while the positive control included 1.6 mg/mL potassium dichromate (Sigma–Aldrich, Johannesburg, South Africa).

Dead brine shrimp were excluded from the mortality calculations by observing the plates under a light microscope (Leica DMi1, 40× magnification) immediately after sample addition (time 0). Mortality assessments were further conducted after 24 h and 48 h. Following the final observation, 50 μL of glacial acetic acid (100% *v/v*; Saarchem, Johannesburg, South Africa) was added to each well to determine the final mortality. Samples causing greater than 50% mortality were classified as toxic [32,33,35,36].

## 5. Conclusions

Synergistic (5.8% plant/antibiotic; 1.7% plant/antifungal) and non-toxic combinations require further *in vivo* interactive validation. Only then can combination therapy be promoted in a clinical setting; alternatively, combinations can be transitioned into formulation studies. The three concerning toxicity results for plant/antimicrobial combinations require further dose–response analysis to determine toxic doses. The considerable antagonism of 54.6% for plant/antibiotic combinations and 58.6% for plant/antifungal combinations found in this study raises concerns for patient safety if considered for use clinically. Furthermore, a similar prevalence of antagonism for plant/antimicrobial combinations was reported in our previous study with JHB plants. This demonstrates a wider distribution of antagonism, which potentially translates into adverse clinical effects and treatment failure among patients receiving both traditional and conventional antimicrobial therapies [6]. While these plant materials are being sold in an informal market, where there is no South African Health Products Regulatory Authority (SAHPRA) regulation of these product sales in South Africa, attention should be given to stricter guidelines for use in future. Consultation between traders and authoritative bodies could be implemented to advise against concurrent use with prescription drugs.

The findings from this study, therefore, serve to inform future research, aiming to discern the potential risks posed by antagonistic interactions. Furthermore, this study sheds light on possible beneficial synergistic interactions, offering valuable insights into later formulation studies aimed at addressing the ongoing antimicrobial resistance crisis.

## Figures and Tables

**Table 1:** Interactive antagonistic plant/amoxicillin combinations.

Plant	Pathogen	MIC ^1^	MIC ^2^	ΣFIC
Organic extract antagonism				
*Artemisia afra*	*E. faecium*	0.50	0.16	17.09
*Berchemia discolor*		0.50	0.16	17.09
*Elaeodendron transvaalense*		0.50	0.16	17.25
*Gunnera perpensa*	*E. faecium*	0.50	0.16	17.09
*Hydnora africana*		0.50	0.16	17.09
*Jatropha zeyheri*		0.50	0.16	17.09
*Lippia javanica*		0.50	0.16	17.09
*Rapanea melanophloeos*		0.50	0.16	17.09
*Rauvolfia caffra*		0.50	0.16	17.09
*Rhoicissus tridentata*		0.50	0.16	17.01
*Sansevieria hyacinthoides*		1.50	0.47	50.76
*Senecio serratuloides*		0.50	0.16	17.09
*Strychnos henningsii*		1.00	0.31	33.89
*Warburgia salutaris*		0.50	0.16	17.09
*Artemisia afra*	*S. aureus*	2.00	0.63	34.53
*Berchemia discolor*		4.00	1.25	68.62
*Elaeodendron transvaalense*		1.00	0.63	36.28
*Rapanea melanophloeos*		2.00	0.63	34.97
*Sansevieria hyacinthoides*		4.00	1.25	67.96
*Senecio oxyriifolius*		4.00	1.25	68.62
*Senecio serratuloides*		1.00	0.31	17.16
*Strychnos henningsii*		4.00	1.25	67.96
*Warburgia salutaris*		1.00	0.31	17.35
*Artemisia afra*	*A. baumannii*	1.00	0.31	34.98
*Berchemia discolor*		1.00	0.31	34.45
*Callilepis laureola*		1.00	0.31	34.16
*Elaeodendron transvaalense*		1.00	0.31	36.79
*Gunnera perpensa*		2.00	0.63	70.07
*Hydnora africana*		2.00	0.63	68.75
*Jatropha zeyheri*		2.00	0.63	68.75
*Lippia javanica*		1.50	0.47	51.50
*Rapanea melanophloeos*		1.00	0.31	34.65
*Rauvolfia caffra*		2.00	0.63	68.42
*Rhoicissus tridentata*		2.00	0.63	68.75
*Sansevieria hyacinthoides*		2.00	0.63	68.75
*Senecio oxyriifolius*		2.00	0.63	68.75
*Senecio serratuloides*		2.00	0.63	70.07
*Strychnos henningsii*		2.00	0.63	68.75
*Warburgia salutaris*		2.00	0.63	68.42
*Gunnera perpensa*	*P. aeruginosa*	2.00	0.63	4.63
*Artemisia afra*	*E. cloacae*	4.00	1.25	19.40
*Senecio serratuloides*		1.50	0.47	7.48
Aqueous extract antagonism				
*Acorus calamus*	*K. pneumoniae*	4.00	1.25	134.60
*Sansevieria hyacinthoides*		3.00	0.94	101.00
*Senecio serratuloides*		4.00	1.25	135.49
*Hydnora africana*	*A. baumannii*	1.50	0.47	50.76
*Rauvolfia caffra*		1.50	0.47	50.76
*Senecio serratuloides*		1.00	0.31	33.86

^1^ MIC for plant extract (mg/mL). ^2^ MIC for amoxicillin (µg/mL).

**Table 2:** Interactive antagonistic plant/azithromycin combinations.

Plant	Pathogen	MIC ^1^	MIC ^2^	ΣFIC
Organic extract antagonism				
*Artemisia afra*	*E. faecium*	4.00	1.25	4.11
*Hydnora africana*		4.00	1.25	4.11
*Senecio serratuloides*		4.00	1.25	4.11
*Warburgia salutaris*		4.00	1.25	4.11
*Artemisia afra*	*S. aureus*	4.00	1.25	4.54
*Berchemia discolor*	*K. pneumoniae*	1.50	0.47	5.79
*Elaeodendron transvaalense*		1.00	0.31	4.24
*Warburgia salutaris*		1.50	0.47	5.38
*Artemisia afra*	*A. baumannii*	2.00	0.63	6.73
*Elaeodendron transvaalense*		1.00	0.31	5.18
*Hydnora africana*		4.00	1.25	10.83
*Jatropha zeyherii*		1.50	0.47	4.06
*Lippia javanica*		4.00	1.25	10.83
*Rauvolfia caffra*		2.00	0.63	5.08
*Senecio oxyriifolius*		1.50	0.47	4.10
*Strychnos henningsii*		4.00	1.25	10.83
*Artemisia afra*	*P. aeruginosa*	0.50	0.16	16.73
*Berchemia discolor*		1.00	0.31	34.13
*Callilepis laureola*		2.00	0.63	66.28
*Elaeodendron transvaalense*		0.75	0.24	25.94
*Gunnera perpensa*		1.00	0.31	34.39
*Hydnora africana*		0.38	0.12	12.80
*Jatropha zeyherii*		0.50	0.16	16.83
*Lippia javanica*		0.75	0.24	25.12
*Rapanea melanophloeos*		0.50	0.16	16.80
*Rauvolfia caffra*		0.75	0.24	25.00
*Rhoicissus tridentata*		1.50	0.47	49.99
*Sansevieria hyacinthoides*		1.00	0.31	33.14
*Senecio oxyriifolius*		2.00	0.63	65.95
*Senecio serratuloides*		0.50	0.16	16.80
*Strychnos henningsii*		1.50	0.47	49.74
*Warburgia salutaris*		1.50	0.47	49.84
Aqueous extract antagonism				
*Callilepis laureola*	*A. baumannii*	3.00	0.94	6.65
*Artemisia afra*	*P. aeruginosa*	2.00	0.63	66.28
*Berchemia discolor*		0.50	0.16	16.48
*Elaeodendron transvaalense*		0.50	0.16	16.73
Aqueous extract antagonism				
*Gunnera perpensa*	*P. aeruginosa*	0.50	0.16	16.48
*Lippia javanica*		4.00	1.25	131.91
*Rapanea melanophloeos*		1.00	0.31	33.07
*Rauvolfia caffra*		4.00	1.25	131.91
*Senecio serratuloides*		0.50	0.16	16.52
*Strychnos henningsii*		4.00	1.25	131.90
Organic extract synergism				
*Callilepis laureola*	*S. aureus*	0.25	0.08	0.26
*Elaeodendron transvaalense*		0.25	0.08	0.50
*Hydnora africana*		0.50	0.16	0.50
*Jatropha zeyherii*		0.25	0.08	0.22
*Senecio serratuloides*		0.50	0.16	0.50
*Berchemia discolor*	*E. cloacae*	0.25	0.08	0.53
*Elaeodendron transvaalense*		0.25	0.08	0.33
Aqueous extract synergism				
*Berchemia discolor*	*E. cloacae*	0.38	0.12	0.49
*Gunnera perpensa*		0.50	0.16	0.36

^1^ MIC for plant extract (mg/mL). ^2^ MIC for azithromycin (µg/mL).

**Table 3:** Interactive antagonistic plant/ciprofloxacin combinations.

Plant	Pathogen	MIC ^1^	MIC ^2^	ΣFIC
Organic extract antagonism				
*Jatropha zeyheri*	*E. faecium*	3.00	0.94	102.01
*Lippia javanica*		4.00	1.25	135.93
*Strychnos henningsii*		1.00	0.31	33.86
*Warburgia salutaris*		4.00	1.25	135.93
*Acorus calamus*	*S. aureus*	4.00	1.25	5.25
*Artemisia afra*		3.00	0.94	51.83
*Berchemia discolor*		4.00	1.25	68.62
*Callilepis laureola*		4.00	1.25	68.62
*Elaeodendron transvaalense*		4.00	1.25	72.56
*Gunnera perpensa*		4.00	1.25	70.31
*Jatropha zeyheri*		4.00	1.25	68.06
*Lippia javanica*		3.00	0.94	51.50
*Rapanea melanophloeos*		1.00	0.94	52.48
*Sansevieria hyacinthoides*		4.00	1.25	67.96
*Senecio oxyriifolius*		4.00	1.25	68.62
*Senecio serratuloides*	*S. aureus*	4.00	1.25	68.62
*Strychnos henningsii*		4.00	1.25	67.96
*Warburgia salutaris*		4.00	1.25	69.41
*Elaeodendron transvaalense*	*K. pneumoniae*	0.38	0.12	13.02
*Lippia javanica*		0.50	0.16	17.01
*Rapanea melanophloeos*		0.25	0.08	8.47
*Rauvolfia caffra*		0.25	0.08	8.47
*Senecio serratuloides*		4.00	1.25	135.27
*Warburgia salutaris*		4.00	1.25	135.49
*Artemisia afra*	*A. baumannii*	2.00	0.63	70.07
*Berchemia discolor*		4.00	1.25	137.76
*Callilepis laureola*		1.00	0.31	34.16
*Elaeodendron transvaalense*		1.00	0.31	36.79
*Hydnora africana*		4.00	1.25	137.24
*Jatropha zeyheri*		1.00	0.31	34.32
*Lippia javanica*		1.50	0.47	51.50
*Rapanea melanophloeos*		2.00	0.63	69.41
*Rauvolfia caffra*		0.50	0.16	17.17
*Rhoicissus tridentata*		2.00	0.63	68.75
*Sansevieria hyacinthoides*		1.00	0.31	34.32
*Senecio oxyriifolius*		1.00	0.31	34.32
*Senecio serratuloides*		4.00	1.25	139.87
*Strychnos henningsii*		2.00	0.63	68.75
*Warburgia salutaris*		4.00	1.25	136.58
*Artemisia afra*	*P. aeruginosa*	4.00	1.25	5.54
Aqueous extract antagonism				
*Sansevieria hyacinthoides*	*E. faecium*	2.00	0.63	67.31
*Lippia javanica*	*K. pneumoniae*	0.13	0.039	4.35
*Rapanea melanophloeos*		0.38	0.12	12.75
*Rauvolfia caffra*		0.19	0.06	6.45
*Senecio serratuloides*		0.50	0.16	17.03
*Strychnos henningsii*		0.19	0.06	6.44
*Callilepis laureola*	*A. baumannii*	4.00	1.25	135.27
*Rauvolfia caffra*		2.00	0.63	50.76
*Senecio serratuloides*		4.00	1.25	135.40
*Berchemia discolor*	*E. cloacae*	1.50	0.47	7.28
Organic extract synergism				
*Callilepis laureola*	*E. cloacae*	0.90	0.03	0.43
*Strychnos henningsii*	*P. aeruginosa*	0.06	0.02	0.07

^1^ MIC for plant extract (mg/mL). ^2^ MIC for ciprofloxacin (µg/mL).

**Table 4:** Interactive antagonistic and synergistic plant/doxycycline combinations.

Plant	Pathogen	MIC ^1^	MIC ^2^	ΣFIC
Organic extract antagonism				
*Lippia javanica*	*E. faecium*	4.00	1.25	4.11
*Artemisia afra*	*S. aureus*	4.00	1.25	4.54
*Hydnora africana*		4.00	1.25	4.11
*Lippia javanica*		4.00	1.25	4.11
*Senecio oxyriifolius*		4.00	1.25	4.11
*Senecio serratuloides*		4.00	1.25	4.11
*Warburgia salutaris*		4.00	1.25	4.89
*Artemisia afra*	*A. baumannii*	2.00	0.63	13.56
*Elaeodendron transvaalense*		0.50	0.16	4.30
*Gunnera perpensa*		2.00	0.63	13.56
*Hydnora africana*		4.00	1.25	24.50
*Jatropha zeyheri*		4.00	1.25	24.50
*Sansevieria hyacinthoides*		4.00	1.25	24.50
*Senecio oxyriifolius*		4.00	1.25	24.50
*Senecio serratuloides*		1.50	0.47	7.44
*Callilepis laureola*	*P. aeruginosa*	4.00	1.25	4.11
*Elaeodendron transvaalense*		2.00	0.63	4.56
*Jatropha seyheri*		4.00	1.25	5.42
*Rhoicissus tridentata*		4.00	1.25	4.76
*Callilepis laureola*	*E. cloacae*	4.00	1.25	4.11
*Lippia javanica*		4.00	1.25	4.11
*Rauvolfia caffra*		4.00	1.25	4.37
Aqueous extract antagonism				
*Gunnera perpensa*	*K. pneumoniae*	4.00	1.25	13.29
*Callilepis laureola*	*A. baumannii*	3.00	0.94	16.91
*Rauvolfia caffra*		1.50	0.47	8.45
*Artemisia afra*	*P. aeruginosa*	4.00	1.25	4.11
*Berchemia discolor*	*E. cloacae*	4.00	1.25	5.42
*Elaeodendron transvaalense*		4.00	1.25	5.42
Organic extract synergism				
*Artemisia afra*	*E. faecium*	0.50	0.16	0.50
*Rapanea melanophloeos*		0.38	0.12	0.39
*Rapanea melanophloeos*	*K. pneumoniae*	0.38	0.12	0.50
*Berchemia discolor*	*P. aeruginosa*	0.19	0.06	0.38
*Rapanea melanophloeos*	*E. cloacae*	0.25	0.08	0.30
Aqueous extract synergism				
*Gunnera perpensa*	*E. cloacae*	0.50	0.16	0.43
*Rapanea melanophloeos*		0.50	0.16	0.45

^1^ MIC for plant extract (mg/mL). ^2^ MIC for doxycycline (µg/mL).

**Table 5:** Interactive antagonistic plant/nystatin combinations.

Plant	Pathogen	MIC ^1^	MIC ^2^	ΣFIC
Organic extract antagonism				
*Berchemia discolor*	*C. albicans*	1.00	3.13	4.92
*Hydnora africana*		4.00	12.50	19.67
*Jatropha zeyheri*		2.00	6.25	4.33
*Lippia javanica*		1.50	4.69	7.38
*Rapanea melanophloeos*		1.50	4.69	4.28
*Rauvolfia caffra*		1.50	4.69	7.38
*Rhoicissus tridentata*		1.50	4.69	7.38
*Strychnos henningsii*		2.00	6.25	5.71
*Callilepis laureola*	*C. glabrata*	4.00	12.50	11.90
*Gunnera perpensa*		0.50	1.56	7.91
*Lippia javanica*		4.00	12.50	13.22
Aqueous extract antagonism				
*Strychnos henningsii*	*C. albicans*	4.00	12.50	5.23
*Lippia javanica*	*C. glabrata*	4.00	12.50	4.70
*Senecio hyacinthoides*		4.00	12.50	4.70
*Strychnos henningsii*		4.00	12.50	4.70

^1^ MIC for plant extract (mg/mL). ^2^ MIC for nystatin (µg/mL).

**Table 6:** Interactive antagonistic and synergistic plant/fluconazole combinations.

Plant	Pathogen	MIC ^1^	MIC ^2^	ΣFIC
Organic extract antagonism				
*Gunnera perpensa*	*C. albicans*	2.00	6.25	6.76
*Jatropha zeyheri*		2.00	6.25	5.39
*Lippia javanica*		1.00	3.13	5.45
*Rhoicissus tridentata*		2.00	6.25	10.89
*Sansevieria hyacinthoides*		2.00	6.25	10.89
*Senecio serratuloides*		2.00	6.25	6.76
*Strychnos henningsii*	*C. albicans*	2.00	6.25	6.76
*Warburgia salutaris*		2.00	6.25	10.89
*Berchemia discolor*	*C. glabrata*	2.00	6.25	9.41
*Elaeodendron transvaalense*		4.00	12.50	14.70
*Hydnora africana*		3.00	9.38	10.00
*Lippia javanica*		2.00	6.25	9.41
*Rapanea melanophloeos*		4.00	12.50	16.08
Organic extract antagonism				
*Rhoicissus tridentata*	*C. glabrata*	4.00	12.50	16.08
*Senecio oxyriifolius*		4.00	12.50	12.64
*Senecio serratuloides*		1.50	4.69	4.74
Aqueous extract antagonism				
*Rapanea melanophloeos*	*C. glabrata*	2.00	6.25	4.07
*Senecio oxyriifolius*		4.00	12.50	4.70
*Senecio serratuloides*		4.00	12.50	4.70
Organic extract synergism				
*Acorus calamus*	*C. albicans*	0.13	0.39	0.34

^1^ MIC for plant extract (mg/mL). ^2^ MIC for fluconazole (µg/mL).

**Table 7:** Toxicity of plant/antimicrobial combinations in the BSLA.

Combination	Plant Sample	Mortality (%) ± S.D. ^1^ at 24 h	Mortality (%) ± S.D. ^1^ at 48 h
Azithromycin + organic extracts	*Acorus calamus*	27.48 ± 8.67	27.48 ± 8.67
	*Callilepis laureola*	4.76 ± 8.25	4.76 ± 8.25
	*Elaeodendron transvaalense*	0.00 ± 0.00	11.76 ± 8.92
	*Hydnora africana*	0.00 ± 0.00	0.00 ± 0.00
Azithromycin + organic extracts	*Jatropha zeyheri*	9.10 ± 5.48	15.62 ± 6.59
	*Rapanea melanophloeos*	0.00 ± 0.00	0.00 ± 0.00
Azithromycin + aqueous extracts	*Berchemia discolor*	0.00 ± 0.00	0.00 ± 0.00
	*Gunnera perpensa*	0.00 ± 0.00	**52.88 ± 8.55**
	*Rapanea melanophloeos*	0.00 ± 0.00	0.00 ± 0.00
Ciprofloxacin + organic extracts	*Callilepis laureola*	0.00 ± 0.00	9.90 ± 13.43
	*Strychnos henningsii*	0.00 ± 0.00	8.50 ± 6.82
Doxycycline + organic extracts	*Artemisia afra*	0.00 ± 0.00	**71.10 ± 11.51**
	*Rapanea melanophloeos*	0.00 ± 0.00	**51.33 ± 25.03**
	*Rauvolfia caffra*	0.45 ± 1.10	2.05 ± 3.41
Doxycycline + aqueous extracts	*Berchemia discolor*	1.03 ± 1.63	15.74 ± 19.32
	*Gunnera perpensa*	0.00 ± 0.00	**57.45 ± 27.08**
Fluconazole + organic extracts	*Acorus calamus*	0.00 ± 0.00	**78.79 ± 28.49**

^1^ standard deviation; bold text shows toxicity. Negative control consisted of saltwater (32 g/L)—0.00 ± 0.00% ± S.D. mortality; positive control consisting of potassium dichromate (1.6 mg/mL)—100.00 ± 0.00% ± S.D. mortality.

## Data Availability

All data generated or analyzed during this study and supporting the conclusions of this article are included in this manuscript. Further data are given in the Supplementary Materials.

## Supplementary Materials

The following supporting information can be downloaded at: https://www.mdpi.com/article/10.3390/antibiotics14050512/s1. Additional files in the form of tables (Tables S1–S4) and figures (Figures S1–S19) can be found in SM file. Table S1: The MIC values of plant extracts (mg/mL) and conventional antibiotics (µg/mL) when tested individually against ESKAPE pathogens; Table S2: MIC values for plant extracts (mg/mL) and conventional antifungals when tested individually against yeasts; Table S3: Average mortality (%) rates in the BSLA of individual plant samples proving synergistic in various combinations; Table S4: Medicinal plants procured from the Warwick traditional medicine market in Durban, KZN. Figure S1: Acorus calamus combined with antibiotics against ESKAPE pathogens; Figure S2: Artemisia afra combined with antibiotics against ESKAPE pathogens; Figure S3: Berchemia discolor combined with antibiotics against ESKAPE pathogens; Figure S4: Callilepis laureola combined with antibiotics against ESKAPE pathogens; Figure S5: Elaeodendron transvaalense combined with antibiotics against ESKAPE pathogens; Figure S6: Gunnera perpensa combined with antibiotics against ESKAPE pathogens; Figure S7: Hydnora africana combined with antibiotics against ESKAPE pathogens; Figure S8: Jatropha zeyheri combined with antibiotics against ESKAPE pathogens; Figure S9: Lippia javanica combined with antibiotics against ESKAPE pathogens; Figure S10: Rapanea melanophloeos combined with antibiotics against ESKAPE pathogens; Figure S11: Rauvolfia caffra combined with antibiotics against ESKAPE pathogens; Figure S12: Rhoicissus tridentata combined with antibiotics against ESKAPE pathogens; Figure S13: Sansevieria hyacinthoides combined with antibiotics against ESKAPE pathogens; Figure S14: Senecio oxyriifolius combined with antibiotics against ESKAPE pathogens; Figure S15: Senecio serratuloides combined with antibiotics against ESKAPE pathogens; Figure S16: Strychnos henningsii combined with antibiotics against ESKAPE pathogens; Figure S17: Warburgia salutaris combined with antibiotics against ESKAPE pathogens; Figure S18: Plant extracts and conventional antifungals against Candida albicans; Figure S19: Plant extracts and conventional antifungals against Candida glabrata.

## Abbreviations

The following abbreviations are used in this manuscript:

MIC	Minimum Inhibitory Concentration
ΣFIC	Sum of the Fractional Inhibitory Concentration
KZN	KwaZulu-Natal
JHB	Johannesburg
SAHPRA	South African Health Products Regulatory Authority

## References

[1] Mander M., Ntuli L., Diederichs N., Mavundla K. (2007). Economics of the traditional medicine trade in South Africa. S. Afr. Health Rev..

[2] World Health Organization (WHO) (2013). WHO Traditional Medicine Strategy (2013–2023).

[3] Dlamini N.S., Darong G.G., Nkwanyana N.M. (2023). South African women’s use of African traditional medicine during pregnancy: A scoping review. Afr. J. Reprod. Health.

[4] Van Wyk B.-E. (2011). The potential of South African plants in the development of new medicinal products. S. Afr. J. Bot..

[5] Bih M.C., Monyama S.L. (2023). South African medicinal plants used in the treatment of human bacterial infections: An updated review. Biomed. Pharmacol. J..

[6] Booth Z., Khumalo G., Van Vuuren S.F. (2024). Toxicity and antimicrobial interactions with conventional antimicrobials of commonly traded medicinal plants from Faraday *muthi* market (Johannesburg, South Africa). S. Afr. J. Bot..

[7] EUCAST (European Committee on Antimicrobial Susceptibility Testing), Clinical Breakpoints—Breakpoints and Guidance. https://www.eucast.org/eucast_news/news_singleview.

[8] Clinical Laboratory Standards Institute (CLSI) (2021). M100—Performance Standards for Antimicrobial Susceptibility Testing.

[9] Booth Z., Van Vuuren S.F., Abia A.L.K., Essack S.Y. (2023). The combined use of African natural products and conventional antimicrobials: An alternative tool against antimicrobial resistance. Antimicrobial Research and One Health in Africa.

[10] EMGuidance Medicines Online Guide. https://emguidance.com.

[11] National Health Service (NHS) Medicines A to Z 2014. https://www.nhs.uk/medicines/.

[12] National Department of Health, South Africa (2019). Essential Drugs Programme. Hospital Level (Adults) Standard Treatment Guidelines and Essential Medicines List. EML Clinical Guideline. 5th ed. https://www.health.gov.za/wp-content/uploads/2024/02/Primary-Healthcare-STGs-and-EML-7th-edition-2020-v3.0.pdf.

[13] (2023). South African Medicines Formulary (SAMF), 14th ed 2023. University of Cape Town. Division of Clinical Pharmacology Eds. South African Medical Association. https://samf-app.com/.

[14] Kim G.Y., Lee C.H. (2015). Antimicrobial susceptibility and pathogenic genes of *Staphylococcus aureus* isolated from the oral cavity of patients with periodontitis. J. Periodontal Implant. Sci..

[15] Ezeh C.K., Eze C.N., Dibua M.E.U., Emencheta C.S. (2023). A meta-analysis on the prevalence of resistance of *Staphylococcus aureus* to different antibiotics in Nigeria. Antimicrob. Resist. Infect. Control..

[16] Watt J.M., Breyer-Brandwijk M.-G. (1962). The Medicinal and Poisonous Plants of Southern and Eastern Africa.

[17] Van Wyk B.-E., Wink M. (2004). Medicinal Plants of the World.

[18] Van Wyk B.-E., Oudthoorn B., Gericke N. (2009). Medicinal Plants of South Africa.

[19] Haile A., Milkessa T. (2022). Antibacterial effects of *Artemisia afra* leaf crude extract against some selected multi-antibiotic resistant clinical pathogens. Ethiop. J. Health Sci..

[20] Del Giudice P. (2020). Skin infections caused by *Staphylococcus aureus*. Acta Derm. Venereol..

[21] Rajput S.B., Karuppayil S.M. (2013). β-asarone, an active principle of *Acorus calamus* rhizome, inhibit morphogenesis, biofilm formation and ergosterol biosynthesis in *Candida albicans*. Phytomedicine.

[22] Phongpaichit S., Pujenjob N., Rukachaisirikul V., Ongsakul M. (2005). Antimicrobial activities of the crude methanol extract of *Acorus calamus* Linn. J. Sci Technol..

[23] Kumar N.S., Aravind S.R., Dileepkimar B.S. (2015). Asarones from *Acorus calamus* in combination with azoles and Amphotericin B: A novel synergistic combination to compete against human pathogenic *Candida* species in vitro. Appl. Biochem. Biotechnol..

[24] Priyadharsini V.J., Girjia A.S., Paramasivan A. (2018). An insight into the emergence of *Acinetobacter baumannii* as an oro-dental pathogen and its drug resistance gene profile—An in-silico approach. Heliyon.

[25] Kumar A., Nair R., Kumar M., Banerjee A., Chakrabarti A., Rudramurthy S.M., Bagga R., Gaur N.A., Mondal A.K. (2020). Assessment of antifungal resistance and associated molecular mechanism in *Candida albicans* isolates from different cohorts of patients in North Indian state of Haryana. Folia Microbiol..

[26] Hodgson T.A., Rachanis C.C. (2002). Oral fungal and bacterial infections in HIV-infected individuals: An overview in Africa. Oral Dis..

[27] United Nations AIDS (UNAIDS) UNAIDS Country Reports 2009. http://www.unaids.org/en/regionscountries/countries/chad/.

[28] Kolisa Y.M., Yengopal V., Shumba K., Igumbor J. (2019). The burden of oral conditions among adolescents living with HIV at a clinic in Johannesburg, South Africa. PLoS ONE.

[29] Mullick J.B., Majumdar T., Ray J., Sil S.K. (2015). Changing trends of *Candida* isolates and their antifungal susceptibility pattern in vulvovaginal candidiasis cases of Tripura, Northeast India. J. Evol. Med. Dent. Sci..

[30] Kaur R., Dhakad M.S., Goyal R., Kumar R. (2016). Emergence of non-albicans *Candida* species and antifungal resistance in intensive care unit patients. Asian Pac. J. Trop. Biomed..

[31] Sanches M.D., Mimura L.A.N., Oliveira L.R.C., Ishikawa L.L.W., Garces H.G., Bagagli  E., Sartori  A., Kurokawa  C.S., Fraga-Silva T.F. (2018). Differential behaviour of non-albicans Candida species in the central nervous system of immunocompetent and immunosuppressed mice. Front. Microbiol..

[32] Chi H.W., Yang Y.S., Shang S.T., Chen K.-E., Yeh K.-M., Chang F.-Y. (2011). *Candida albicans* versus non-albicans bloodstream infections: The comparison of risk factors and outcome. J. Microbiol. Immunol. Infect..

[33] World Health Organization (WHO) Bacterial Pathogens of Public Health Importance to Guide Research, Development, and Strategies to Prevent, and Control Antimicrobial Resistance. The WHO Bacterial Priority Pathogens List..

[34] Van Vuuren S.F., Viljoen A. (2011). Plant-based antimicrobial studies—Methods and approaches to study the interactions between natural products. Planta Medica.

[35] Cock I.E., Kalt F.R. (2010). Toxicity evaluation of *Xanthorrhoea johnsonii* leaf methanolic extract using the *Artemia franciscana* bioassay. Pharmacogn. Mag..

[36] Bussmann R.W., Malca G., Glenn A., Sharon D., Nilsen B., Parris B., Dubose  D., Ruiz  D., Saleda  J., Martinez  M. (2011). Toxicity of medicinal plants used in traditional medicine in Northern Peru. J. Ethnopharmacol..

