# Supplementary material for: Conventional Antimicrobial and Medicinal Plants from a Traditional Medicine Market in South Africa: An Interactive Antimicrobial and Toxicity Study
Source: Antibiotics (Basel). 2025 May 15;14(5):512. doi: 10.3390/antibiotics14050512 (PMC12108390; doi:10.3390/antibiotics14050512)
Supplement: Supplementary file 1 [file antibiotics-14-00512-s001.zip › antibiotics-3585780-supplementary.pdf]

Supplementary Materials

Table S1. The MIC values of plant extracts (mg/ml) and conventional antibiotics (μg/ml) when tested individually against ESKAPE pathogens.

Plant	*E. faecium* ATCC 27270		*S. aureus* ATCC 25923		*K. pneumoniae* ATCC 13883		*A. baumannii* ATCC 19606		*P. aeruginosa* ATCC 27853		*E. cloacae* NCTC 13406	
	**AE**	**OE**	**AE**	**OE**	**AE**	**OE**	**AE**	**OE**	**AE**	**OE**	**AE**	**OE**
Acorus calamus	>8.0	>8.0	>8.0	4.0	>8.0	>8.0	>8.0	4.0	8.0	>8.0	>8.0	>8.0
Artemisia afra	>8.0	4.0	>8.0	3.0	>8.0	>8.0	>8.0	1.0	4.0	2.0	>8.0	2.0
Berchemia discolor	>8.0	4.0	>8.0	4.0	>8.0	2.7	>8.0	1.7	8.0	1.0	2.0	0.8
Callilepis laureola	>8.0	>8.0	>8.0	4.0	>8.0	>8.0	8.0	2.7	>8.0	4.0	>8.0	4.0
Elaeodendron transvaalense	>8.0	2.0	>8.0	1.0	>8.0	1.5	>8.0	0.4	2.0	0.8	2.0	1.7
Gunnera purpensa	>8.0	4.0	>8.0	1.8	0.5	2.7	8.0	1.0	8.0	0.8	8.0	1.3
Hydnora africana	>8.0	4.0	>8.0	4.0	>8.0	>8.0	8.0	2.0	>8.0	1.7	>8.0	2.7
Jatropha zeyheri	>8.0	4.0	>8.0	7.0	>8.0	>8.0	>8.0	2.0	>8.0	2.0	>8.0	>8.0
Lippia javanica	>8.0	4.0	>8.0	4.0	8.0	8.0	>8.0	2.0	8.0	2.7	>8.0	4.0
Rapanea melanophloeos	>8.0	4.0	>8.0	2.0	8.0	2.0	>8.0	1.3	5.3	1.7	4.0	2.0
Rauvolfia caffra	>8.0	4.0	>8.0	>8.0	6.0	5.3	8.0	2.7	8.0	4.0	8.0	3.3
Rhoicissus tridentata	>8.0	8.0	>8.0	>8.0	>8.0	>8.0	>8.0	2.0	>8.0	2.7	8.0	>8.0
Sansevieria hyacinthoides	>8.0	8.0	>8.0	8.0	>8.0	>8.0	>8.0	2.0	>8.0	4.0	>8.0	>8.0
Senecio oxyriifolius	>8.0	>8.0	>8.0	4.0	8.0	>8.0	>8.0	2.0	>8.0	8.0	>8.0	>8.0
Senecio serratuloides	>8.0	4.0	>8.0	4.0	6.0	8.0	6.7	1.0	5.3	1.7	>8.0	1.7
Strychnos henningsii	>8.0	6.0	>8.0	8.0	8.0	>8.0	>8.0	2.0	8.0	4.0	8.0	8.0
Warburgia salutaris	>8.0	4.0	>8.0	2.5	>8.0	6.0	>8.0	2.7	>8.0	3.3	>8.0	2.0

Antibiotic	*E. faecium* ATCC 27270	*S. aureus* ATCC 25923	*K. pneumoniae* ATCC 13883	*A. baumannii* ATCC 19606	*P. aeruginosa* ATCC 27853	*E. cloacae* NCTC 13406
Amoxicillin	0.04	0.08	0.04	0.04	1.80	0.31
Azithromycin	1.88	1.88	0.39	0.64	0.04	2.50
Ciprofloxacin	0.04	0.08	0.04	0.04	1.80	0.31
Doxycycline	1.88	1.88	1.88	0.24	1.88	1.88

(AE) aqueous extract. (OE) organic extract. EUCAST [7]-CLSI [8] breakpoints: amoxicillin 0.001 – 8.00 μg/ml; azithromycin 0.01 – 4.0 μg/ml; ciprofloxacin 0.001 – 4.0 μg/ml; doxycycline 0.1 – 4.0 μg/ml. Negative control of acetone in water >8.0 mg/ml. Positive control of ciprofloxacin (0.01 mg/ml) 0.04 – 1.8 μg/ml. Culture control >8.0 mg/ml.

Table S2. MIC values for plant extracts (mg/ml) and conventional antifungals when tested individually against yeasts.
(AE) aqueous extract. (OE) organic extract. EUCAST [7]-CLSI [8] breakpoints: fluconazole 0.001 - ≤ 16.00 µg/ml; nystatin 0.13 – 8.00 µg/ml. Negative control of acetone in water

Plant	*C. albicans* ATCC 10231		*C. glabrata* ATCC 90030	
	AE	OE	AE	OE
Acorus calamus	>8.0	3.0	>8.0	6.0
Artemisia afra	>8.0	2.0	>8.0	8.0
Berchemia discolor	>8.0	1.0	>8.0	6.0
Callilepis laureola	>8.0	1.5	>8.0	>8.0
Elaeodendron transvaalense	8.0	2.0	>8.0	3.0
Gunnera purpensa	8.0	1.0	8.0	1.0
Hydnora africana	>8.0	3.0	>8.0	3.0
Jatropha zeyheri	>8.0	1.0	>8.0	1.0
Lippia javanica	8.0	2.0	8.0	2.0
Rapanea melanophloeos	8.0	1.0	8.0	2.0
Rauvolfia caffra	8.0	1.0	8.0	8.0
Rhoicissus tridentata	8.0	2.0	8.0	>8.0
Sansevieria hyacinthoides	>8.0	8.0	>8.0	4.0
Senecio oxyriifolius	>8.0	1.0	>8.0	2.0
Senecio serratuloides	>8.0	2.0	>8.0	2.0
Strychnos henningsii	>8.0	1.0	>8.0	1.0
Warburgia salutaris	>8.0	8.0	>8.0	8.0
Antifungal				
Fluconazole	3.13		1.56	
Nystatin	5.21		6.25	

>8.0 mg/ml. Positive control of nystatin (0.1 mg/ml) 5.21 – 6.25 µg/ml. Culture control >8.0 mg/ml.

Table S3. Average mortality (%) rates in the BSLA of individual plant samples proving synergistic in various combinations.

Extract type	Plant	Average mortality (%) ± S.D. at 24 h	Average mortality (%) ± S.D. at 48 h
Organic extracts	*Acorus calamus*	**100.00 ± 0.00**	**100.00 ± 0.00**
	Artemisia afra	36.11 ± 55.49	45.37 ± 47.82
	Callilepis laureola	0.00 ± 0.00	8.50 ± 9.15
	Elaeodendron transvaalense	**75.38 ± 3.85**	**55.75 ± 3.35**
	Hydnora africana	0.00 ± 0.00	0.00 ± 0.00
	Jatropha zeyheri	7.83 ± 0.61	30.37 ± 10.32
	Rapanea melanophloeos	0.00 ± 0.00	9.38 ± 4.88
	Rauvolfia caffra	0.00 ± 0.00	3.46 ± 3.58
	Senecio serratuloides	0.00 ± 0.00	2.56 ± 4.44
	Strychnos henningsii	0.00 ± 0.00	0.00 ± 0.00
Aqueous extracts	*Berchemia discolor*	0.00 ± 0.00	2.22 ± 3.85
	Gunnera perpensa	0.00 ± 0.00	1.15 ± 1.99
	Rapanea melanophloeos	0.00 ± 0.00	0.00 ± 0.00

Bold values - toxicity observed. Negative control of salt water (32 g/l) (average mortality of 0.0 ± 0.00%). Positive control of potassium dichromate (1.6 mg/ml) (average mortality of 100.0 ± 0.00%).

Table S4. Medicinal plants procured from the Warwick traditional medicine market in Durban, KZN.

Plant species collected	Vernacular and common name	Voucher number	Plant part	Traditional uses for the medicinal plant
Acorus calamus L.	*Ikalimuzi* (Z); flag root, sweet flag (E); *makkalmoes* (A)	ZB306	Roots	Digestive complaints, such as dysentry [16-18].
Artemisia afra Jacq. ex Willd.	*Umhlonyane* (Z); African wormwood (E); *wildeals* (A)	ZB315	Leaves and stems	Respiratory tract infections, gastrointestinal complaints and intestinal worms [16-18].
Berchemia discolor (Klotzsch) Hemsl.	*Uvuka* (Z); brown ivory, bird plum, mountain date, wild almond (Eng); *bruin-ivoor, voëlpruim* (A)	ZB320	Bark	Wounds and other skin infections [16-18].
Callilepis laureola DC.	*Impila* (Z); ox-eye daisy (E)	ZB310	Bark	Gastrointestinal complaints [16-18].
Elaeodendron transvaalense (Burtt Davy) R.H.Archer	*Ingwavuma* (Z); bushveld saffron (E); *bosveld-saffraan, lepelhout* (A)	ZB319	Bark	Gastrointestinal complaints and fever, sexually transmitted infections [16-18].
Gunnera perpensa L.	*Ugobho* (Z); river pumpkin (E); *rivierpampoen* (A)	ZB309	Roots	Urinary tract and skin infections [16-18].
Hydnora africana Thunb.	*Umavumbuka* (Z) jackal food (E); *jakkalskos, bobbejaankos* (A)	ZB308	Bark	Gastrointestinal complaints, skin infections, urinary tract and bladder infections [16-18].
Jatropha zeyheri Sond.	*Ugodide* (Z); *verfbol* (A)	ZB312	Bark	Skin ailments and wounds, sexually transmitted infections, urinary tract infections and eye infections [16-18].
Lippia javanica (Burm.f.) Spreng	*Umsuzwane* (Z); fever tea; fever tree, lemon bush (E); *lemoenbossie* (A)	ZB316	Bark and stems	Respiratory and gastrointestinal infections, skin infections, wounds, malaria [16-18].
Rapanea melanophloeos (L.) Mez	*Umaphipha* (Z); Cape Beech (E); *boekenhout, beukenhout* (A)	ZB321	Bark	Respiratory complaints and stomach conditions [16-18].
Rauvolfia caffra Sond.	*UmHlambamanzi, umKhadluvungu* (Z); quinine tree (E); *kinaboom* (A); *umJelo* (X)	ZB322	Bark	Wounds and other skin infections, gastrointestinal complaints and respiratory infections [16-18].
Rhoicissus tridentata (L.f.) Wild & R.B.Drumm.	*Isinwazi* (Z); bitter grape, Bushman's grape, common forest grape (E); *bitterdruif, bobbejaantou, droog-my-keel* (A)	ZB313	Bark	Gynaecological conditions, gastrointestinal complaints, urinary tract and bladder infections [16-18].
Sansevieria hyacinthoides (L.) Druce	*Isikholokotho* (Z); mother-in-law's tongue (E*); skoonma-se-tong, wildeatel, aambeiwortel* (A)	ZB317	Roots	Intestinal parasites, fever, oral cavity infections and ear infections [16-18].
Senecio oxyriifolius DC.	*Idumbe* (Z); false nasturtium (E); *Kappertjieblaar* (A)	ZB323	Roots	Fever associated with bacterial infections [16-18].

Table S4 continued. Medicinal plants procured from the Warwick traditional medicine market in Durban, KZN.

Plant species collected	Vernacular and common name	Voucher number	Plant part	Traditional uses for the medicinal plant
Senecio serratuloides DC.	*Unsukumbili* (Z); two-day cure (E)	ZB307	Leaves and stems	Respiratory infections, skin infections and wounds, sexually transmitted infections [16-18].
Strychnos henningsii Gilg	*Umqalothi* (Z); red bitterberry, coffee hard pear, hard pear (E); *rooibitterbessie, koffiehardepeer, hardepeer* (A)	ZB318	Bark	Gynaecological complaints, wounds, respiratory tract and oral infections, gastrointestinal infections and parasitic infestation [16-18].
Warburgia salutaris (G.Bertol.) Chiov.	*Isibhaha* (Z); pepperbark tree (E)	ZB311	Bark	Respiratory tract infections, malaria, gastrointestinal complaints, sexually transmitted infections, skin infections [16-18].

A = Afrikaans; E = English; X = Xhosa; Z = Zulu. Plants were purchased from traders by Prof. S.F van Vuuren - PhD and Z. Booth - Master of Pharmacy (Department of Pharmacy and Pharmacology, University of the Witwatersrand, South Africa). Plant identification was conducted by Dr. G Khumalo – PhD (Queen Mary University of London, William Harvey Research Institute

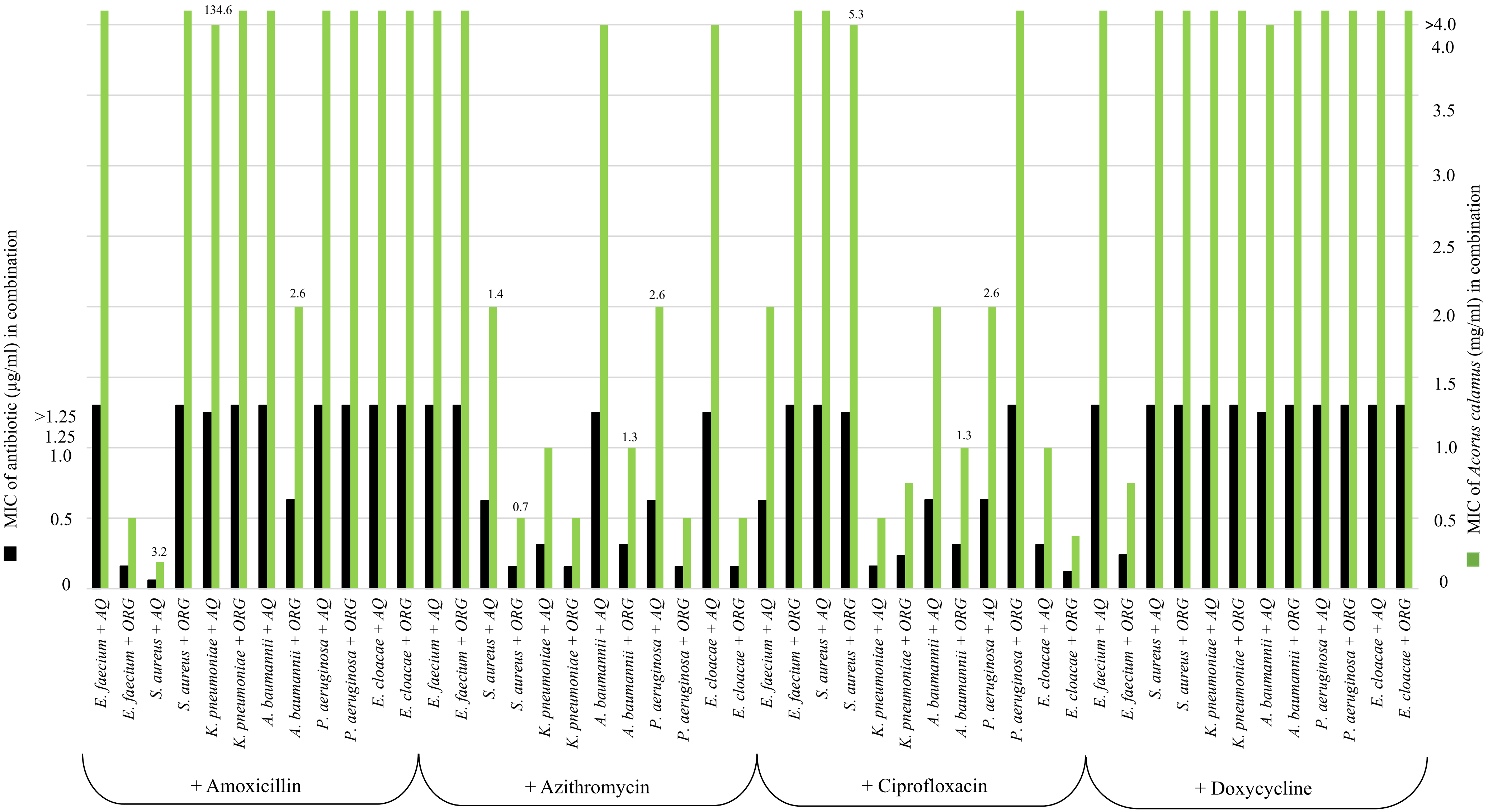

Figure S1. *Acorus calamus* combined with antibiotics against ESKAPE pathogens.

Values indicated above columns ΣFIC could be determined. No value indicated above column ΣFIC could not be determined. (*AQ*) aqueous extract. (*ORG*) organic extract. Acetone in water (32.0 mg/ml) as negative control (MIC >8.0 mg/ml). Ciprofloxacin (0.01 mg.ml) as positive control (MIC 0.039 – 1.800 μg/ml). Culture in TSB as the culture control (MIC >8.0 mg/ml).

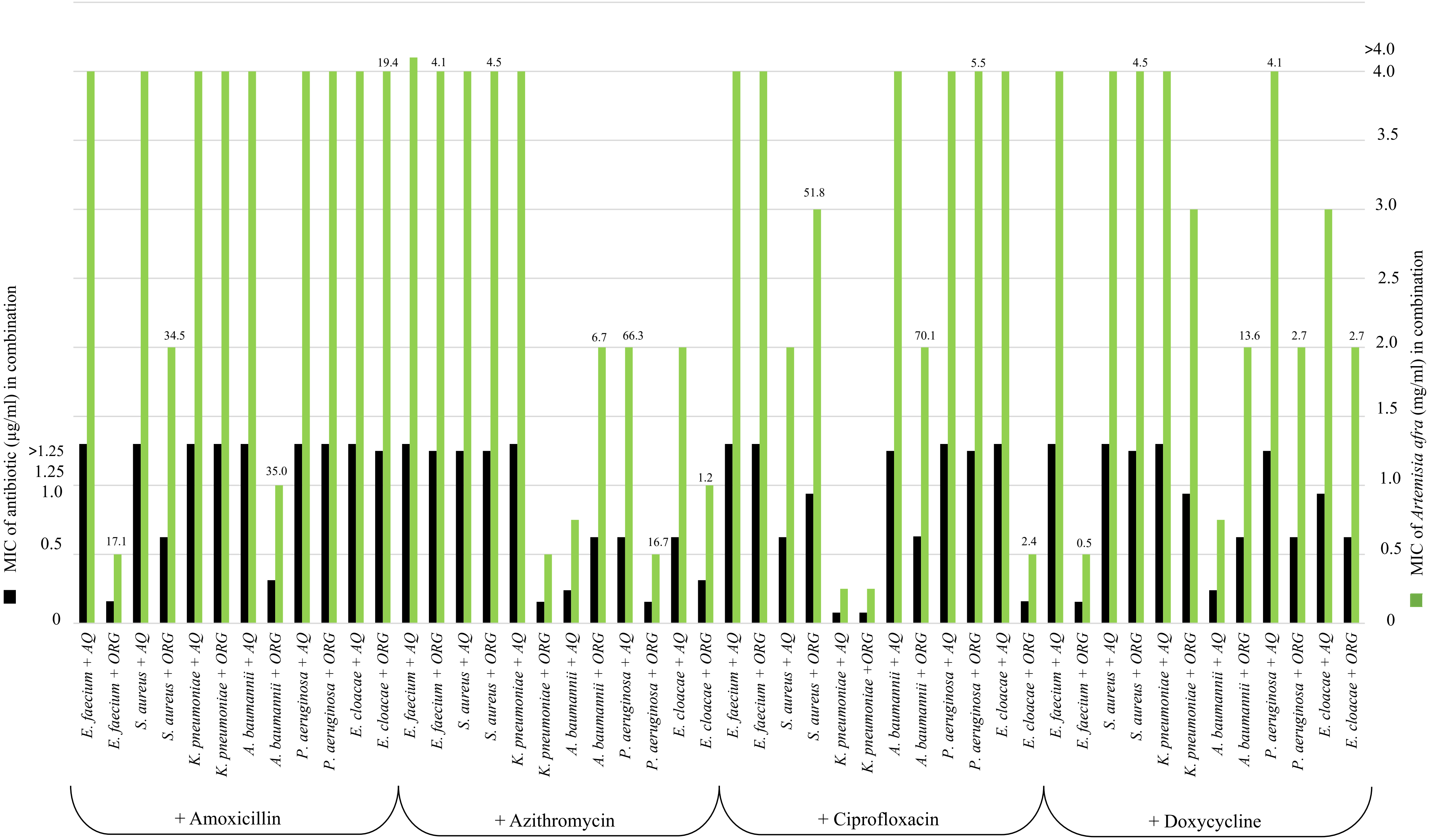

Figure S2. *Artemisia afra* combined with antibiotics against ESKAPE pathogens.

Values indicated above columns ΣFIC could be determined. No value indicated above column ΣFIC could not be determined. (*AQ*) aqueous extract. (*ORG*) organic extract. Acetone in water (32.0 mg/ml) as negative control (MIC >8.0 mg/ml). Ciprofloxacin (0.01 mg.ml) as positive control (MIC 0.039 – 1.800 µg/ml). Culture in TSB as the culture control (MIC >8.0 mg/ml).

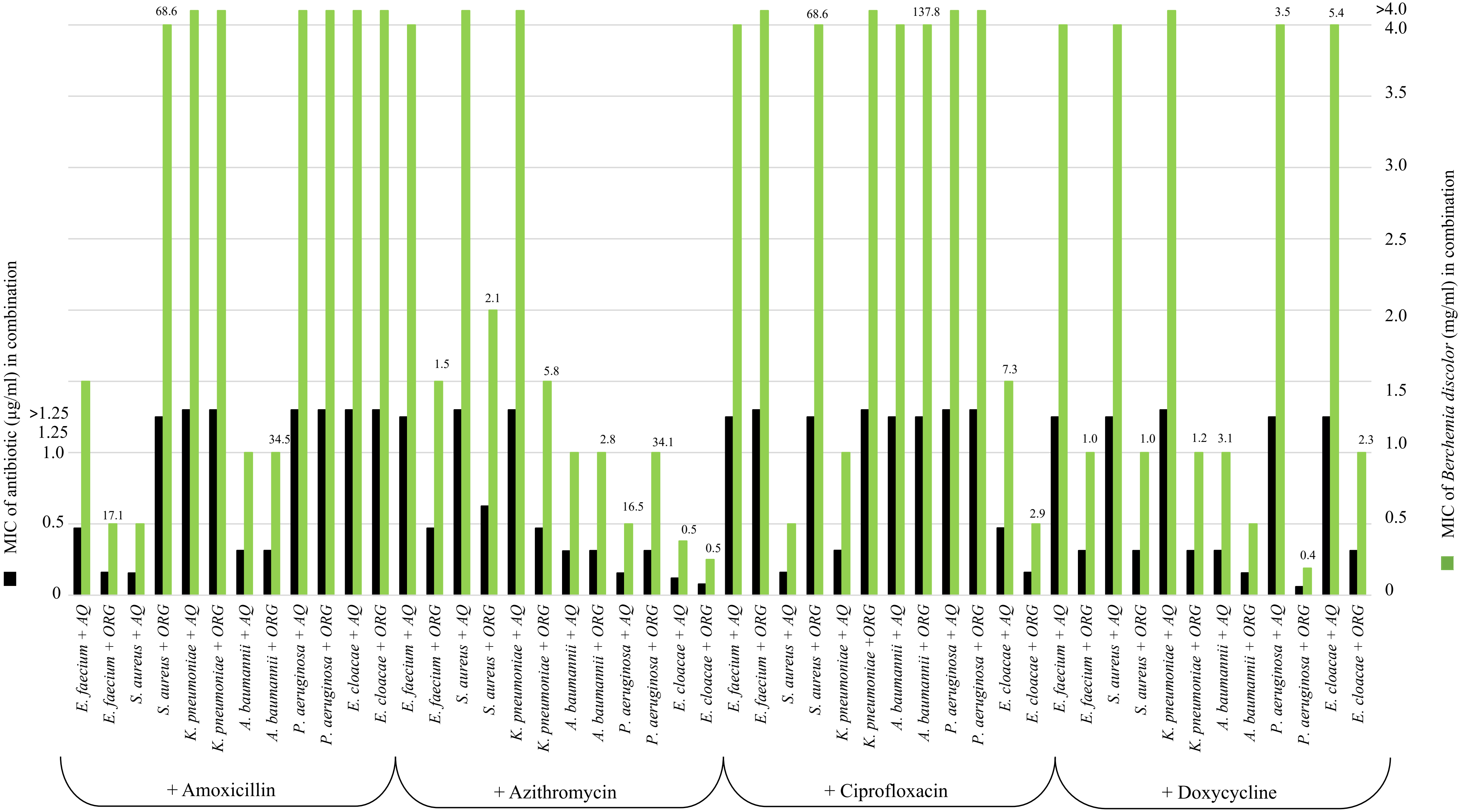

Figure S3. *Berchemia discolor* combined with antibiotics against ESKAPE pathogens.

Values indicated above columns ΣFIC could be determined. No value indicated above column ΣFIC could not be determined. (*AQ*) aqueous extract. (*ORG*) organic extract. Acetone in water (32.0 mg/ml) as negative control (MIC >8.0 mg/ml). Ciprofloxacin (0.01 mg.ml) as positive control (MIC 0.039 – 1.800 µg/ml). Culture in TSB as the culture control (MIC >8.0 mg/ml).

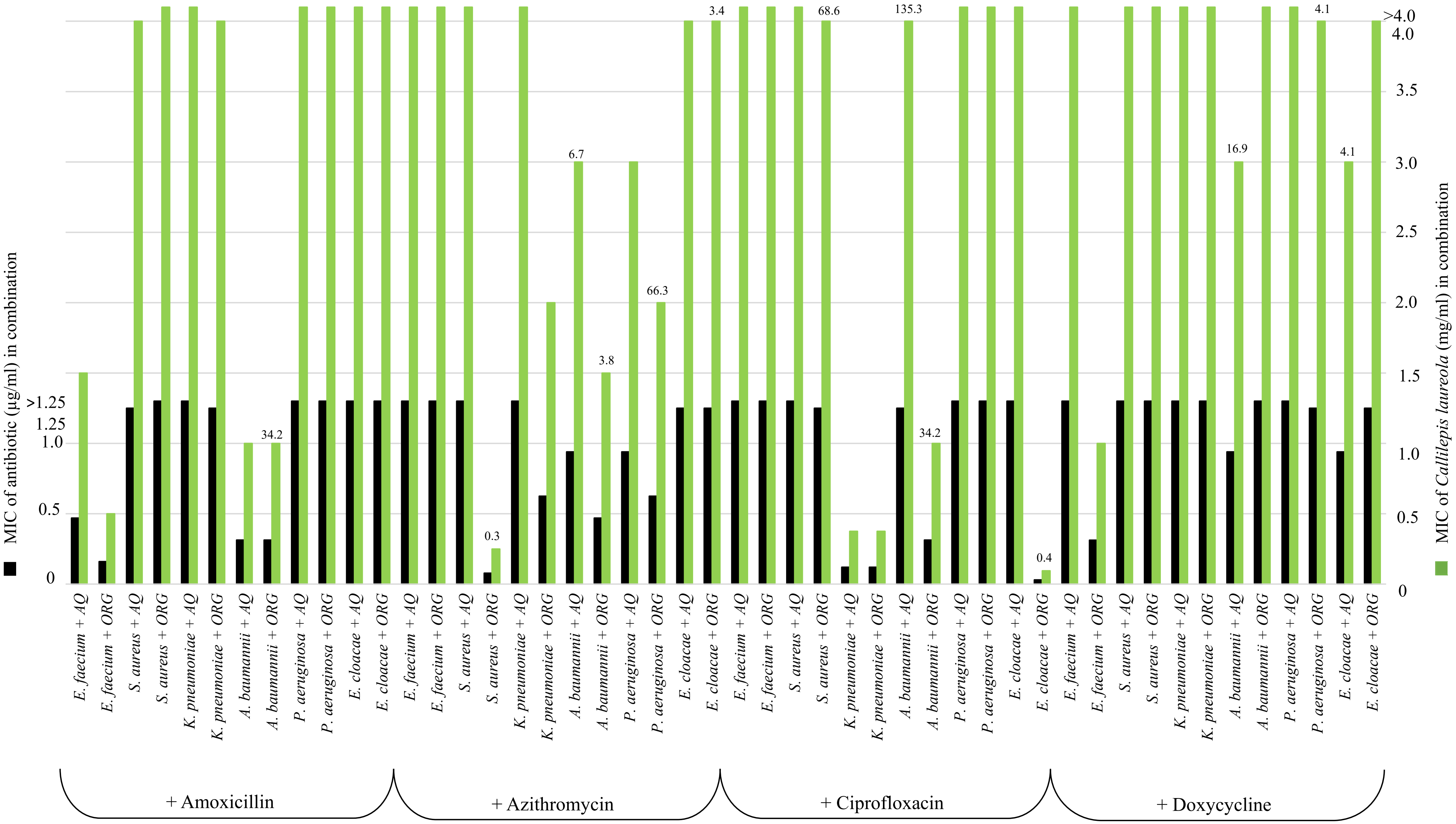

Figure S4. *Callilepis laureola* combined with antibiotics against ESKAPE pathogens.

Values indicated above columns ΣFIC could be determined. No value indicated above column ΣFIC could not be determined. (*AQ*) aqueous extract. (*ORG*) organic extract. Acetone in water (32.0 mg/ml) as negative control (MIC >8.0 mg/ml). Ciprofloxacin (0.01 mg.ml) as positive control (MIC 0.039 – 1.800 µg/ml). Culture in TSB as the culture control (MIC >8.0 mg/ml).

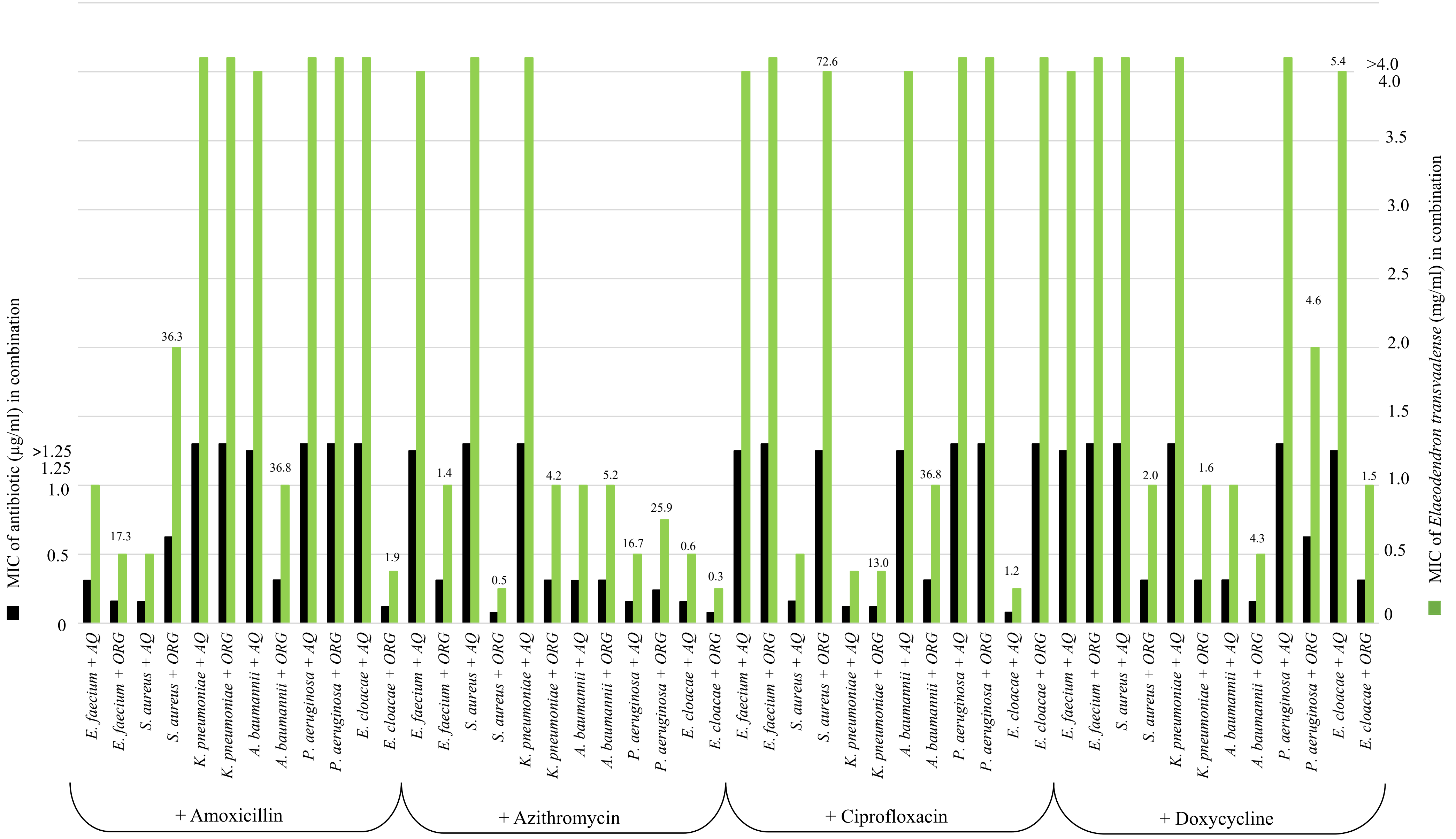

Figure S5. *Elaeodendron transvaalense* combined with antibiotics against ESKAPE pathogens.

Values indicated above columns ΣFIC could be determined. No value indicated above column ΣFIC could not be determined. (*AQ*) aqueous extract. (*ORG*) organic extract. Acetone in water (32.0 mg/ml) as negative control (MIC >8.0 mg/ml). Ciprofloxacin (0.01 mg.ml) as positive control (MIC 0.039 – 1.800 µg/ml). Culture in TSB as the culture control (MIC >8.0 mg/ml).

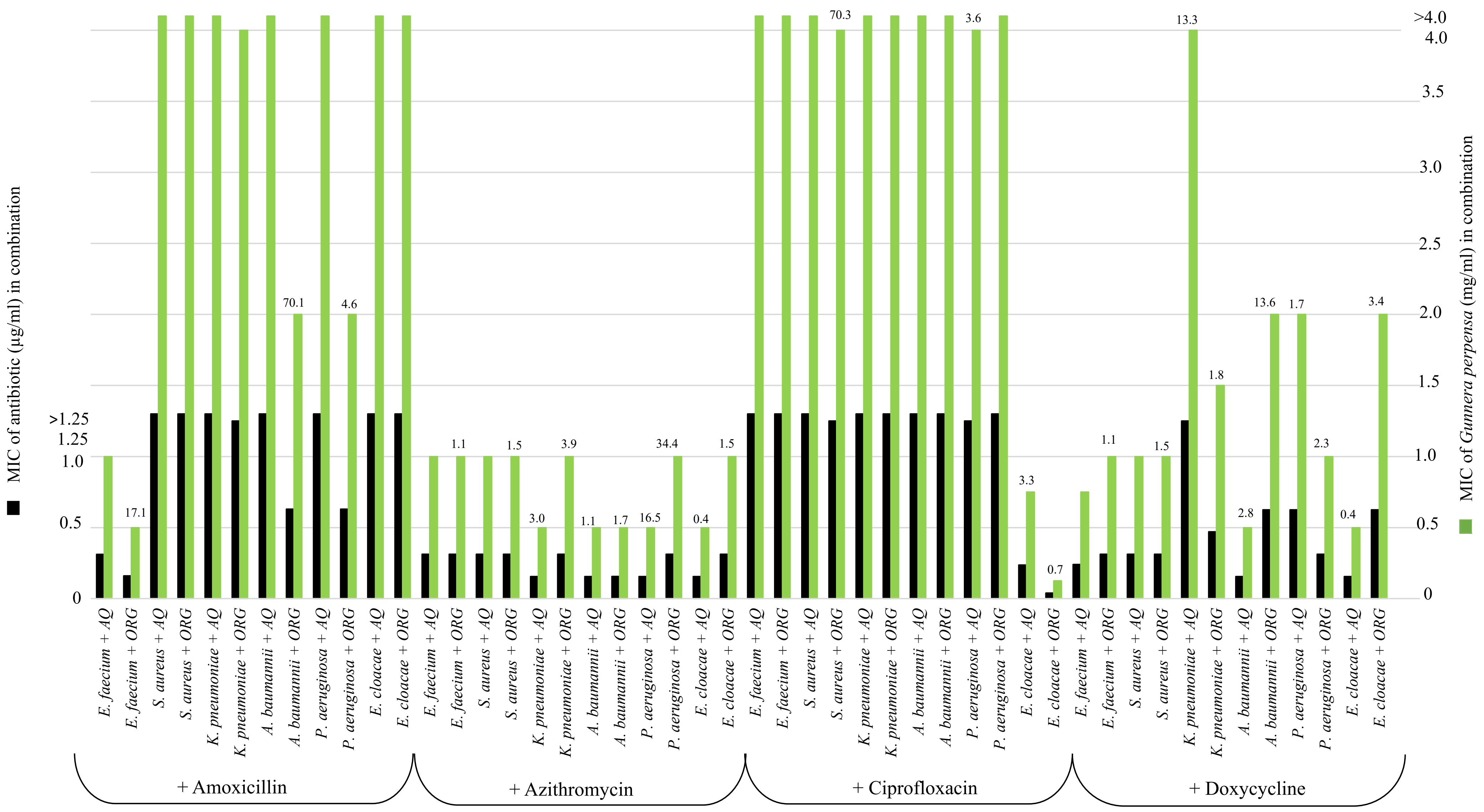

Figure S6. *Gunnera perpensa* combined with antibiotics against ESKAPE pathogens.

Values indicated above columns ΣFIC could be determined. No value indicated above column ΣFIC could not be determined. (*AQ*) aqueous extract. (*ORG*) organic extract. Acetone in water (32.0 mg/ml) as negative control (MIC >8.0 mg/ml). Ciprofloxacin (0.01 mg.ml) as positive control (MIC 0.039 – 1.800 µg/ml). Culture in TSB as the culture control (MIC >8.0 mg/ml).

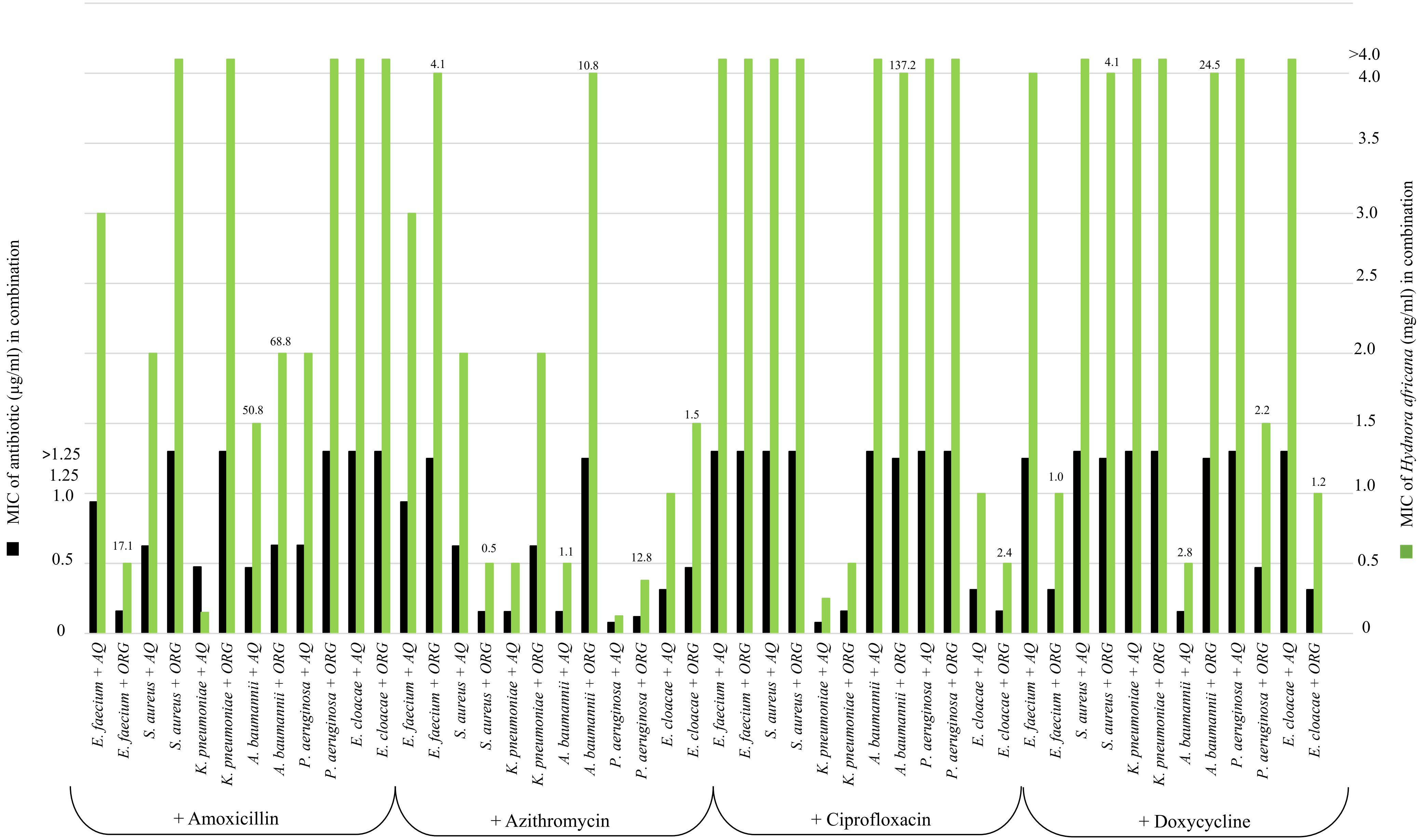

Figure S7. *Hydnora africana* combined with antibiotics against ESKAPE pathogens.

Values indicated above columns ΣFIC could be determined. No value indicated above column ΣFIC could not be determined. (*AQ*) aqueous extract. (*ORG*) organic extract. Acetone in water (32.0 mg/ml) as negative control (MIC >8.0 mg/ml). Ciprofloxacin (0.01 mg.ml) as positive control (MIC 0.039 – 1.800 µg/ml). Culture in TSB as the culture control (MIC >8.0 mg/ml).

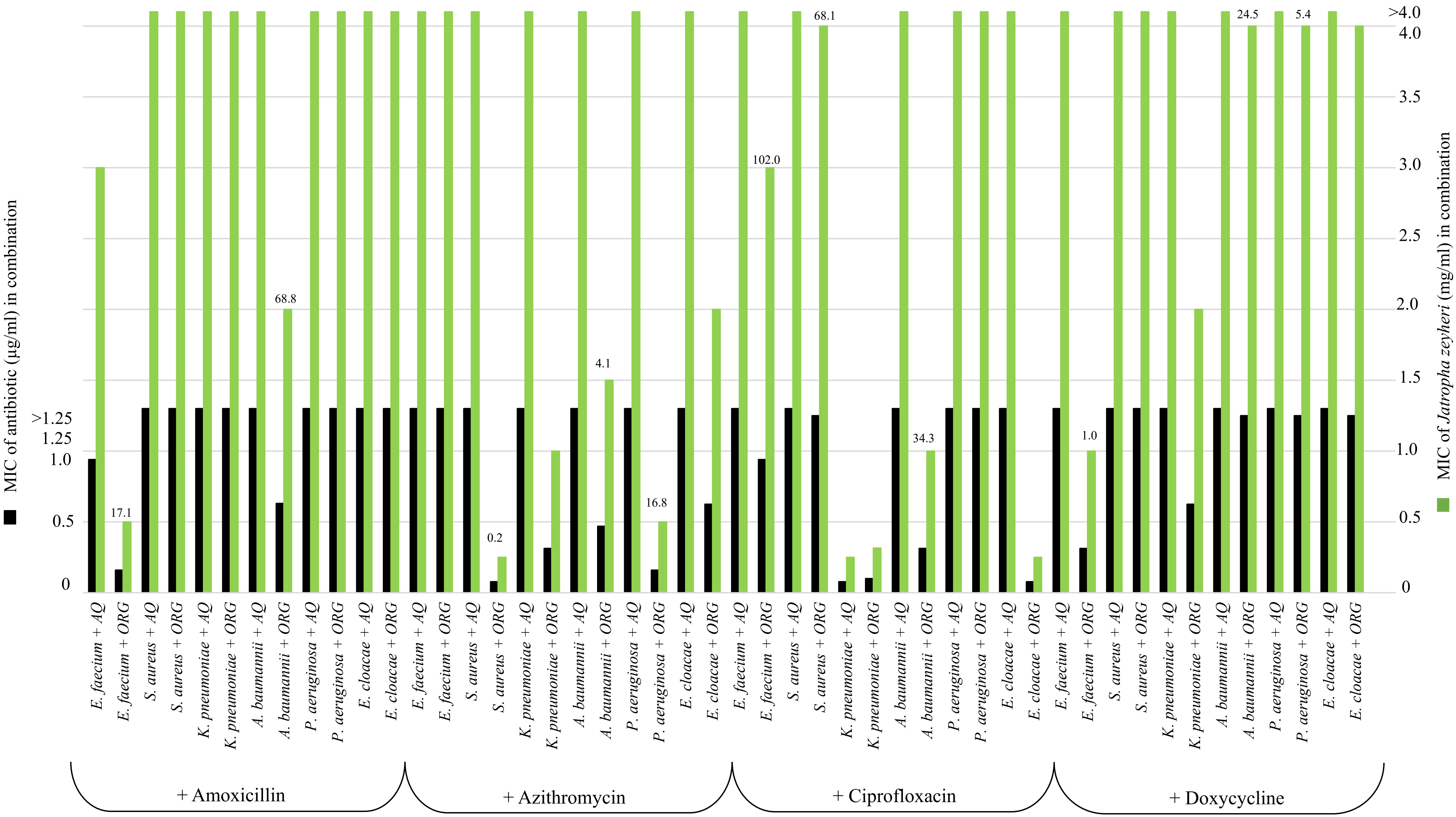

Figure S8. *Jatropha zeyheri* combined with antibiotics against ESKAPE pathogens.

Values indicated above columns ΣFIC could be determined. No value indicated above column ΣFIC could not be determined. (*AQ*) aqueous extract. (*ORG*) organic extract. Acetone in water (32.0 mg/ml) as negative control (MIC >8.0 mg/ml). Ciprofloxacin (0.01 mg.ml) as positive control (MIC 0.039 – 1.800 µg/ml). Culture in TSB as the culture control (MIC >8.0 mg/ml).

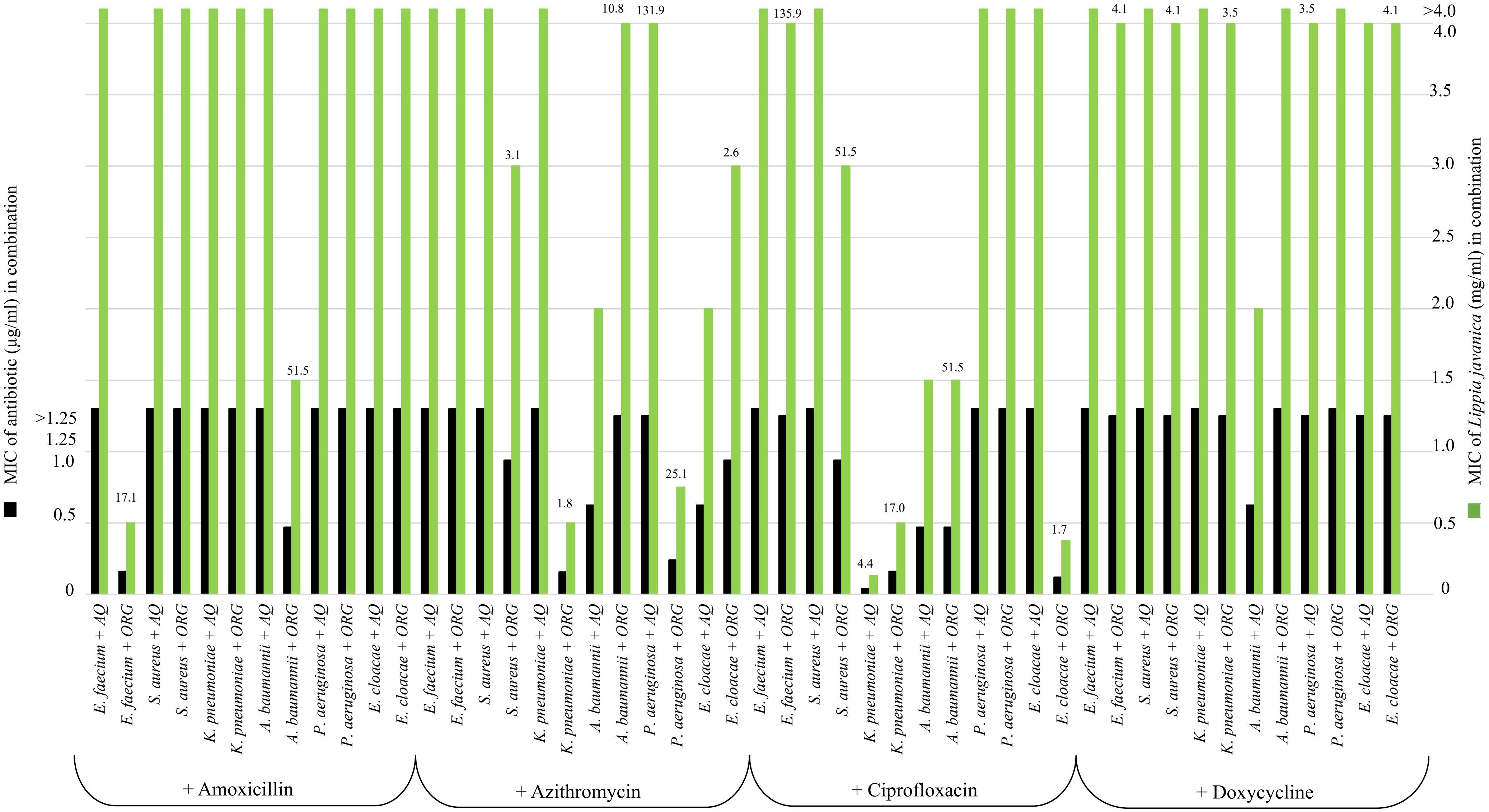

Figure S9. *Lippia javanica* combined with antibiotics against ESKAPE pathogens.

Values indicated above columns ΣFIC could be determined. No value indicated above column ΣFIC could not be determined. (*AQ*) aqueous extract. (*ORG*) organic extract. Acetone in water (32.0 mg/ml) as negative control (MIC >8.0 mg/ml). Ciprofloxacin (0.01 mg.ml) as positive control (MIC 0.039 – 1.800 µg/ml). Culture in TSB as the culture control (MIC >8.0 mg/ml).

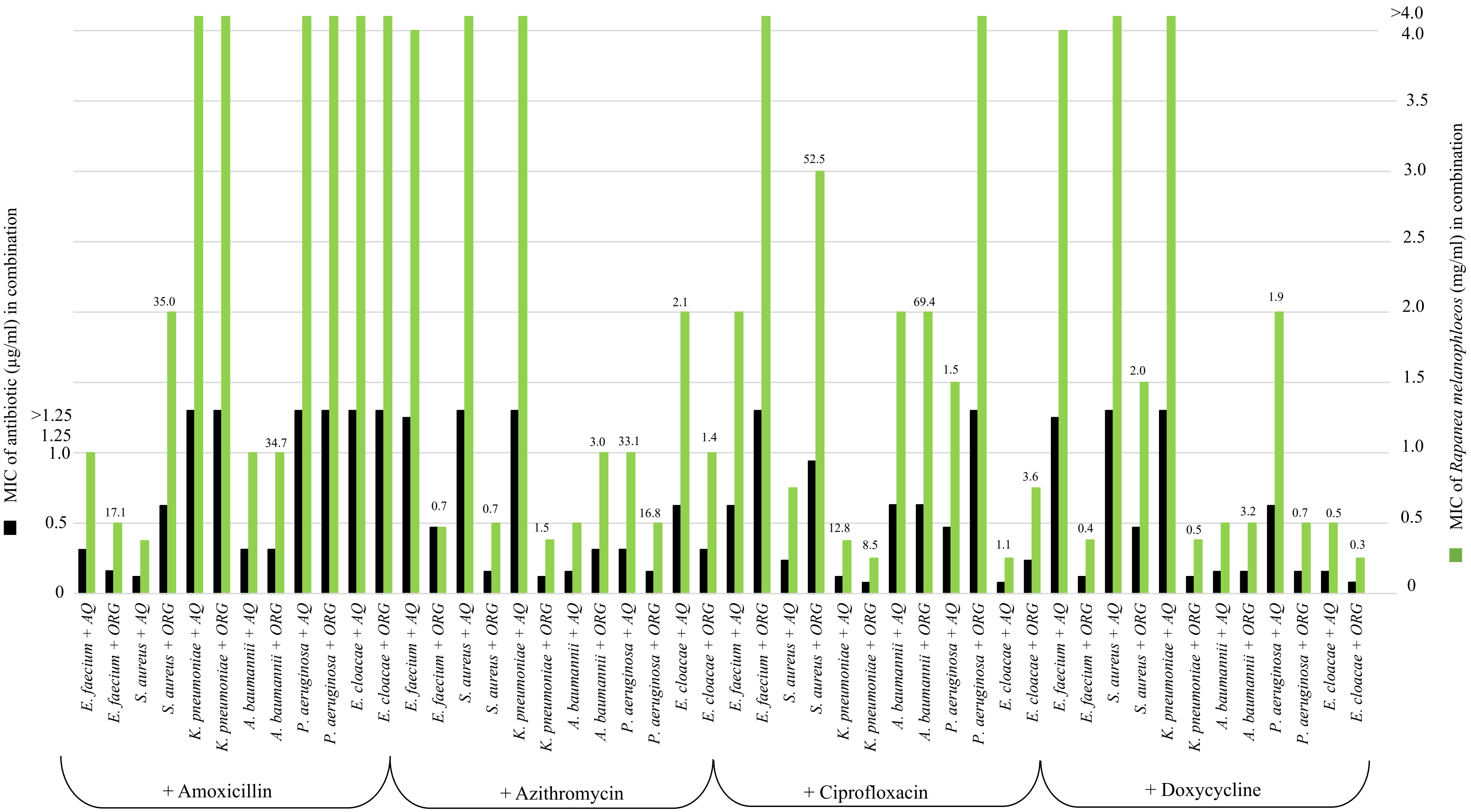

Figure S10. *Rapanea melanophloeos* combined with antibiotics against ESKAPE pathogens.

Values indicated above columns ΣFIC could be determined. No value indicated above column ΣFIC could not be determined. (*AQ*) aqueous extract. (*ORG*) organic extract. Acetone in water (32.0 mg/ml) as negative control (MIC >8.0 mg/ml). Ciprofloxacin (0.01 mg.ml) as positive control (MIC 0.039 – 1.800 µg/ml). Culture in TSB as the culture control (MIC >8.0 mg/ml).

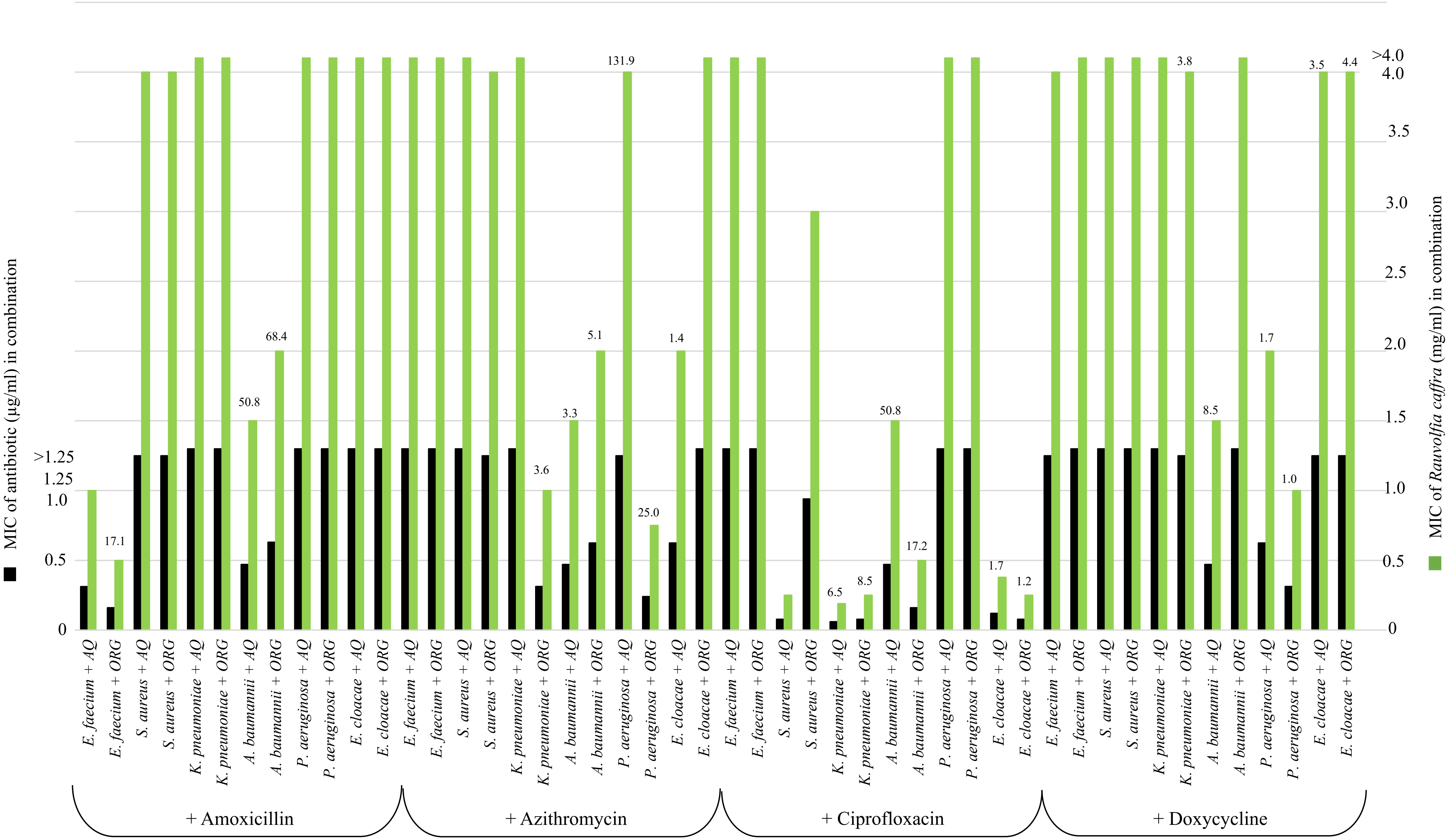

Figure S11. *Rauvolfia caffra* combined with antibiotics against ESKAPE pathogens.

Values indicated above columns ΣFIC could be determined. No value indicated above column ΣFIC could not be determined. (*AQ*) aqueous extract. (*ORG*) organic extract. Acetone in water (32.0 mg/ml) as negative control (MIC >8.0 mg/ml). Ciprofloxacin (0.01 mg.ml) as positive control (MIC 0.039 – 1.800 µg/ml). Culture in TSB as the culture control (MIC >8.0 mg/ml).

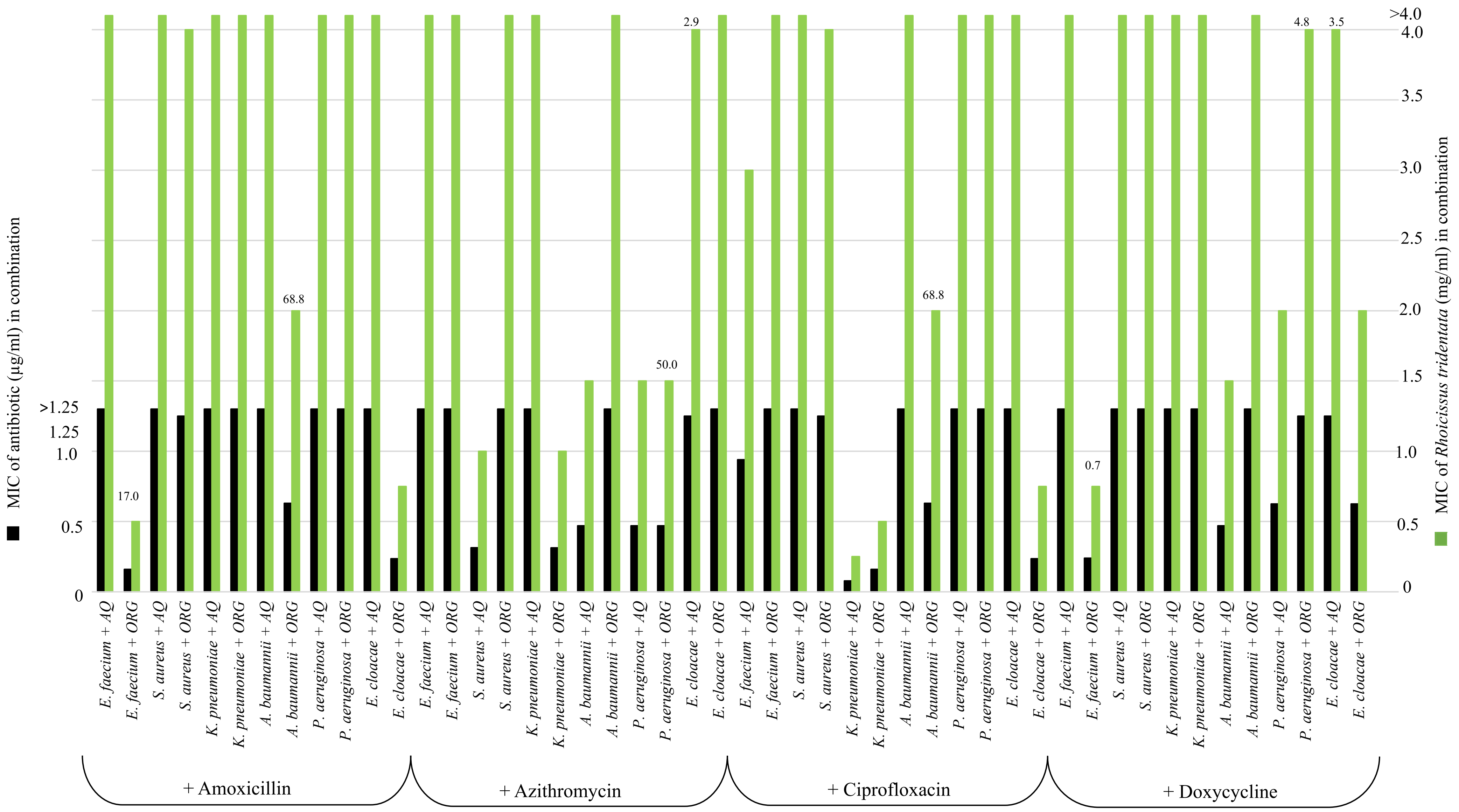

Figure S12. *Rhoicissus tridentata* combined with antibiotics against ESKAPE pathogens.

Values indicated above columns ΣFIC could be determined. No value indicated above column ΣFIC could not be determined. (*AQ*) aqueous extract. (*ORG*) organic extract. Acetone in water (32.0 mg/ml) as negative control (MIC >8.0 mg/ml). Ciprofloxacin (0.01 mg.ml) as positive control (MIC 0.039 – 1.800 µg/ml). Culture in TSB as the culture control (MIC >8.0 mg/ml).

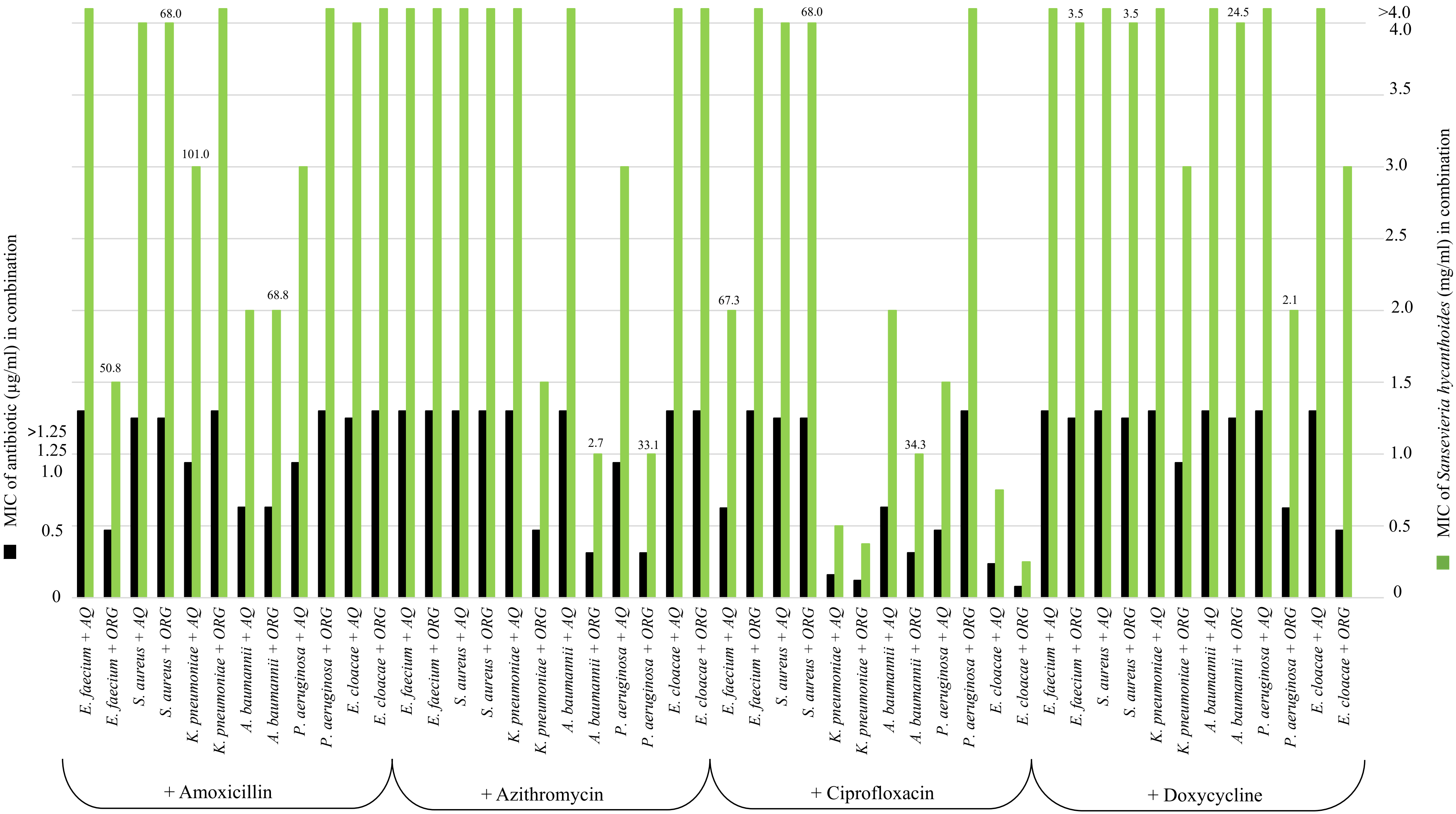

Figure S13. *Sansevieria hyacinthoides* combined with antibiotics against ESKAPE pathogens.

Values indicated above columns ΣFIC could be determined. No value indicated above column ΣFIC could not be determined. (*AQ*) aqueous extract. (*ORG*) organic extract. Acetone in water (32.0 mg/ml) as negative control (MIC >8.0 mg/ml). Ciprofloxacin (0.01 mg.ml) as positive control (MIC 0.039 – 1.800 µg/ml). Culture in TSB as the culture control (MIC >8.0 mg/ml).

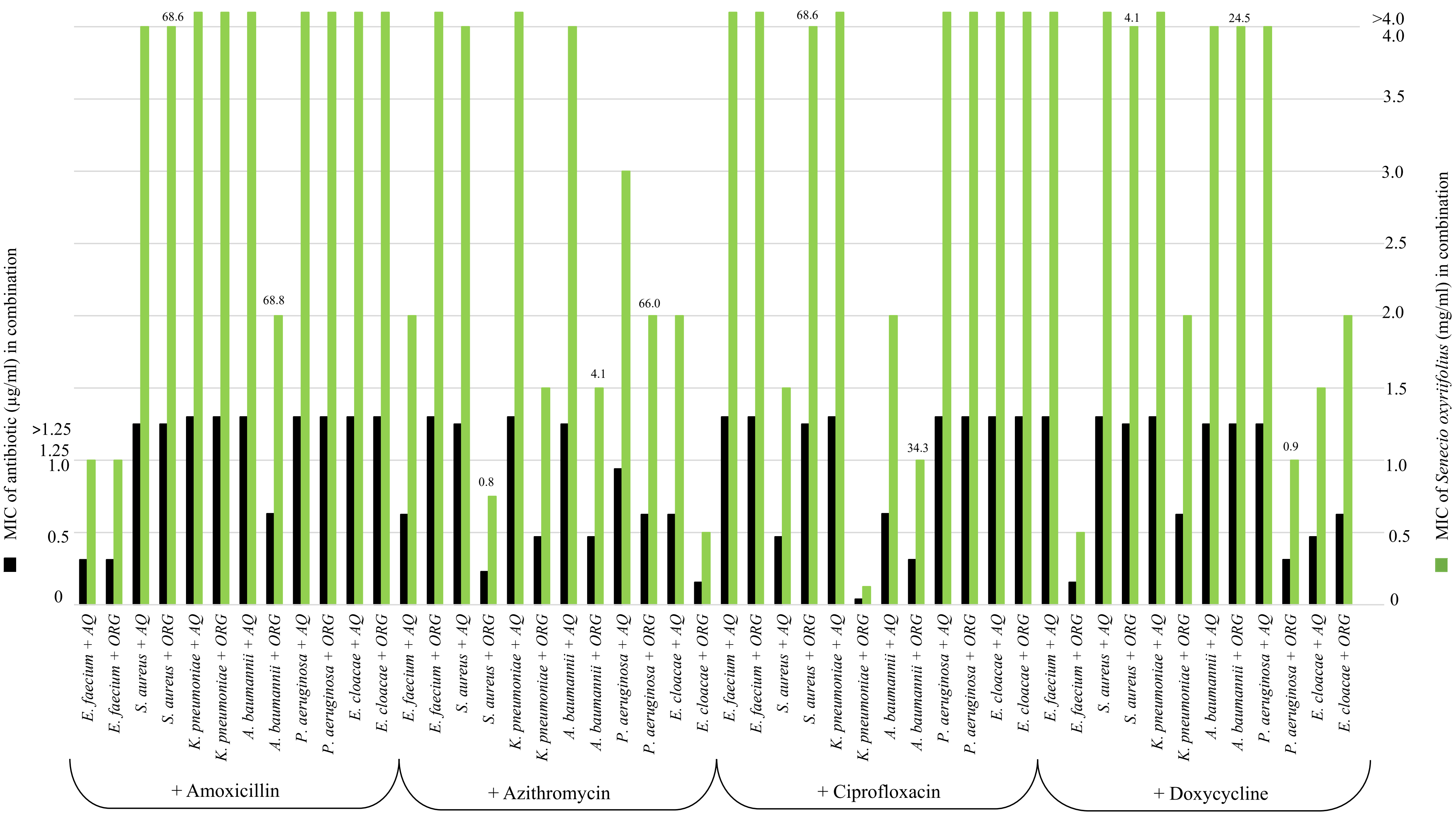

Figure S14. *Senecio oxyriifolius* combined with antibiotics against ESKAPE pathogens.

Values indicated above columns ΣFIC could be determined. No value indicated above column ΣFIC could not be determined. (*AQ*) aqueous extract. (*ORG*) organic extract. Acetone in water (32.0 mg/ml) as negative control (MIC >8.0 mg/ml). Ciprofloxacin (0.01 mg.ml) as positive control (MIC 0.039 – 1.800 µg/ml). Culture in TSB as the culture control (MIC >8.0 mg/ml).

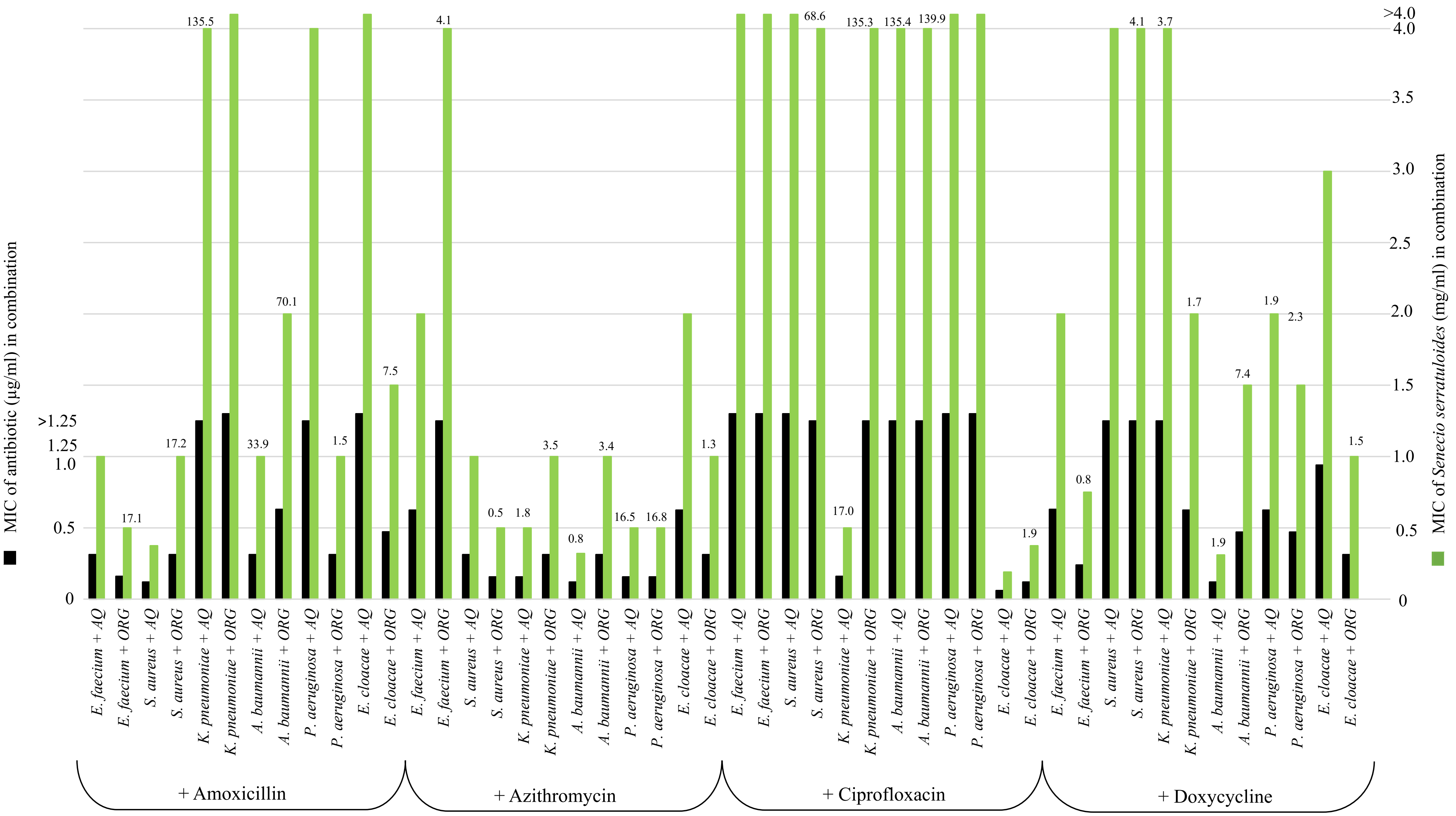

Figure S15. *Senecio serratuloides* combined with antibiotics against ESKAPE pathogens

Values indicated above columns ΣFIC could be determined. No value indicated above column ΣFIC could not be determined. (*AQ*) aqueous extract. (*ORG*) organic extract. Acetone in water (32.0 mg/ml) as negative control (MIC >8.0 mg/ml). Ciprofloxacin (0.01 mg.ml) as positive control (MIC 0.039 – 1.800 µg/ml). Culture in TSB as the culture control (MIC >8.0 mg/ml).

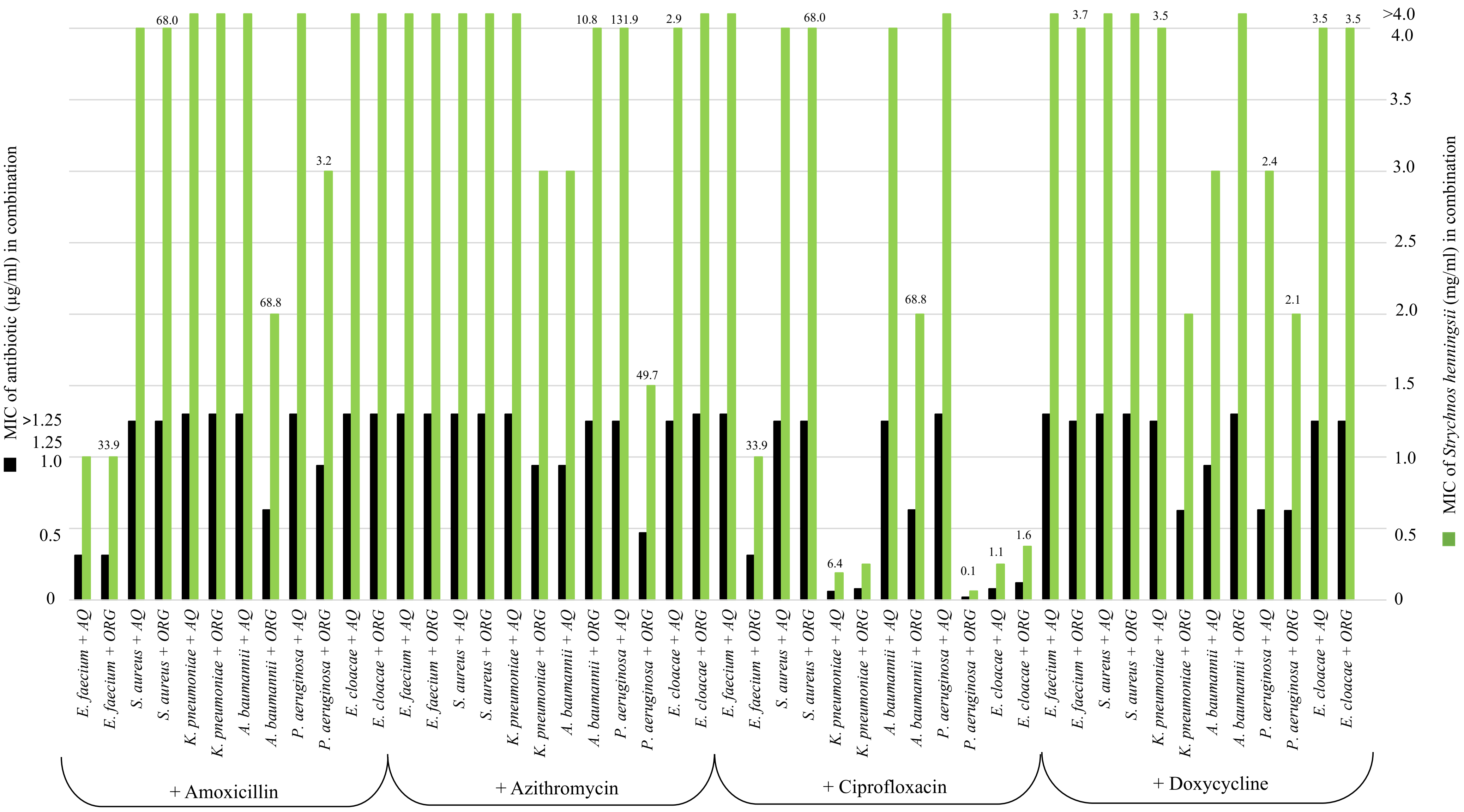

Figure S16. *Strychnos henningsii* combined with antibiotics against ESKAPE pathogens.

Values indicated above columns ΣFIC could be determined. No value indicated above column ΣFIC could not be determined. (*AQ*) aqueous extract. (*ORG*) organic extract. Acetone in water (32.0 mg/ml) as negative control (MIC >8.0 mg/ml). Ciprofloxacin (0.01 mg.ml) as positive control (MIC 0.039 – 1.800 µg/ml). Culture in TSB as the culture control (MIC >8.0 mg/ml).

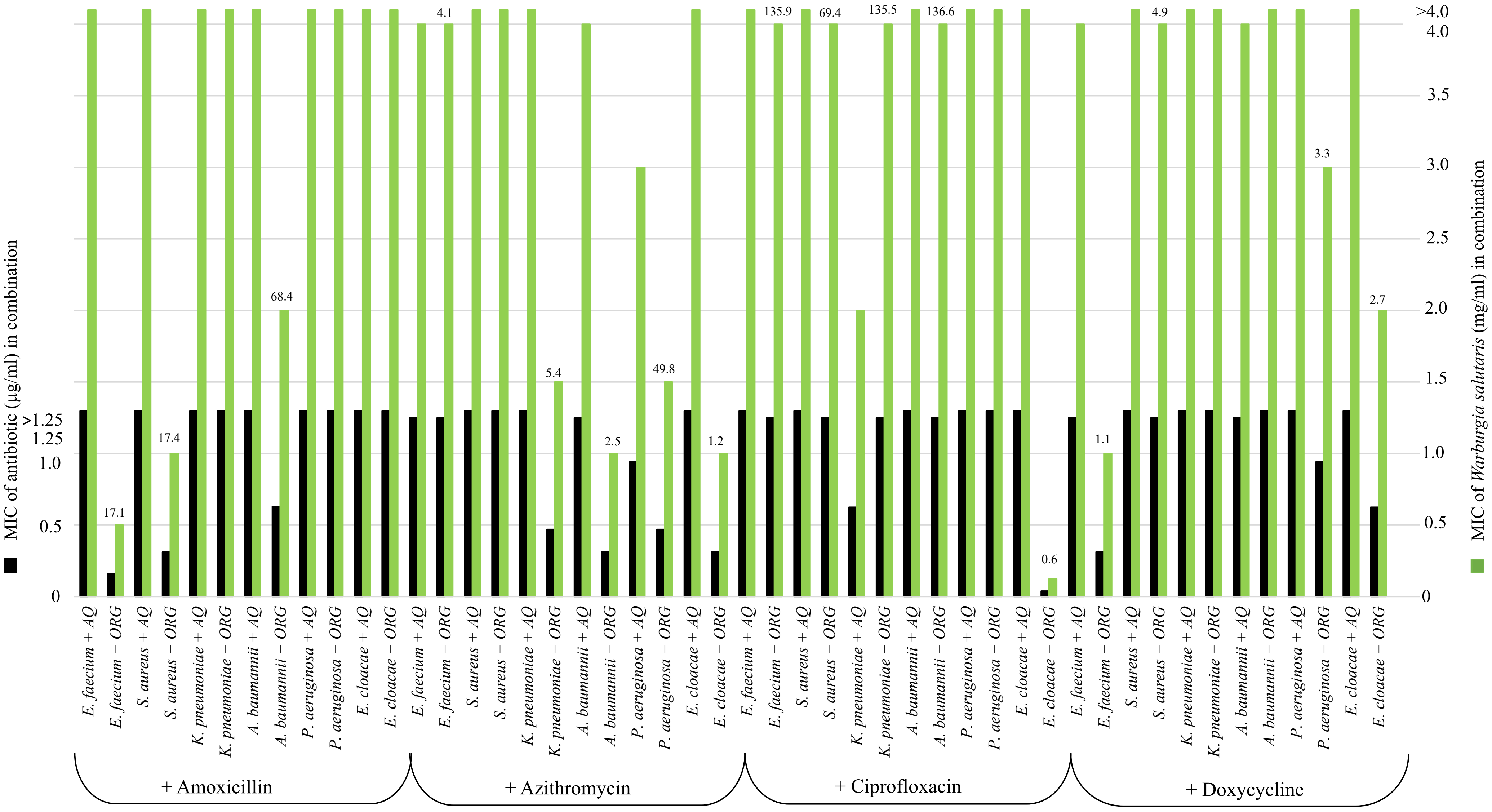

Figure S17. *Warburgia salutaris* combined with antibiotics against ESKAPE pathogens.

Values indicated above columns ΣFIC could be determined. No value indicated above column ΣFIC could not be determined. (*AQ*) aqueous extract. (*ORG*) organic extract. Acetone in water (32.0 mg/ml) as negative control (MIC >8.0 mg/ml). Ciprofloxacin (0.01 mg.ml) as positive control (MIC 0.039 – 1.800 µg/ml). Culture in TSB as the culture control (MIC >8.0 mg/ml).

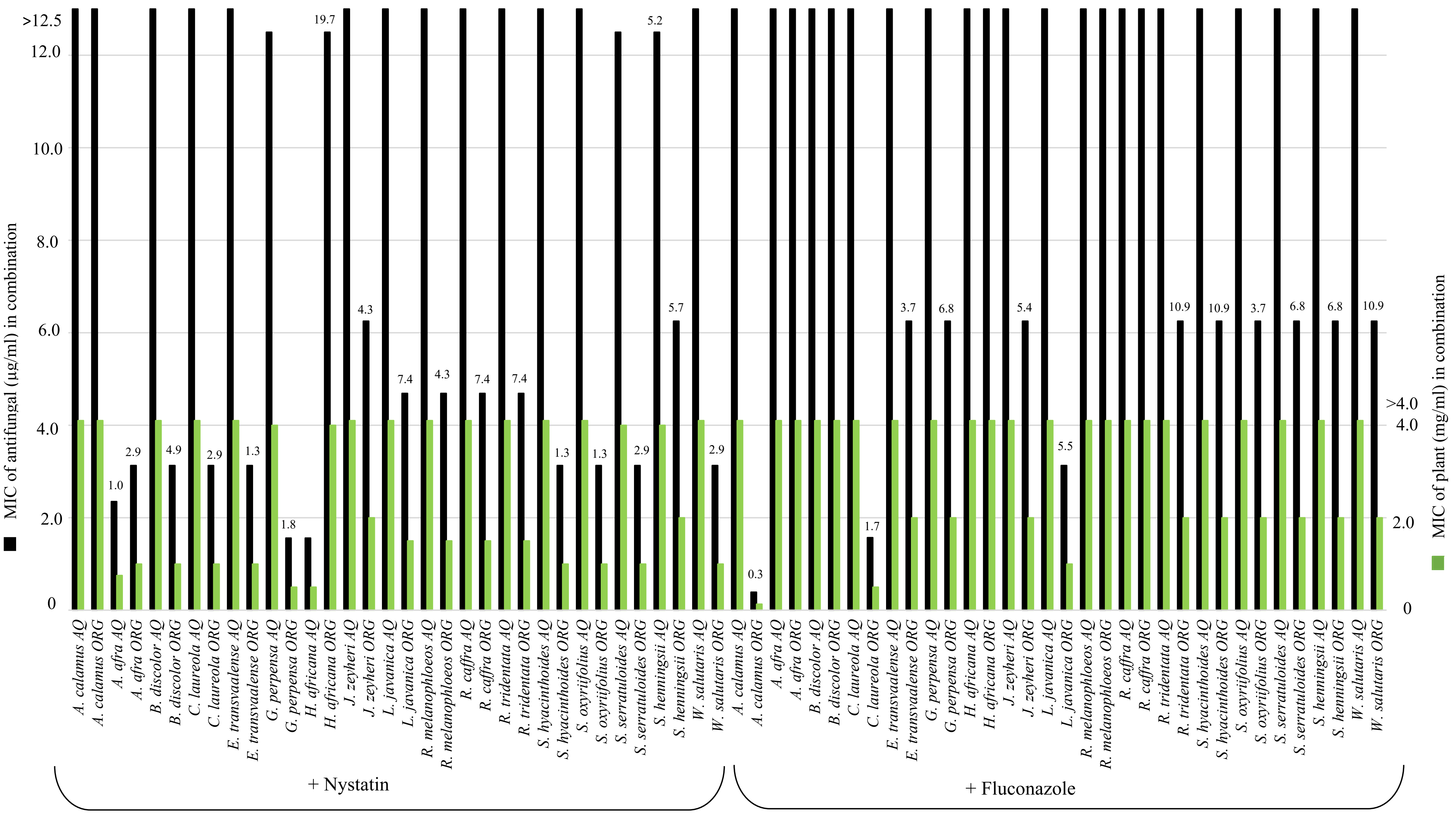

Figure S18. Plant extracts and conventional antifungals against *Candida albicans*.

Individual MIC values in tables as mg/ml. Values indicated above columns ΣFIC could be determined. No value indicated above column ΣFIC could not be determined. (*AQ*) aqueous extract. (*ORG*) organic extract. Acetone in water (32.0 mg/ml) as the negative control (MIC >8.0 mg/ml). Nystatin (0.1 mg/ml) as the positive control (MIC 5.210 µg/ml). Culture in TSB as the culture control (MIC >8.0 mg/ml).

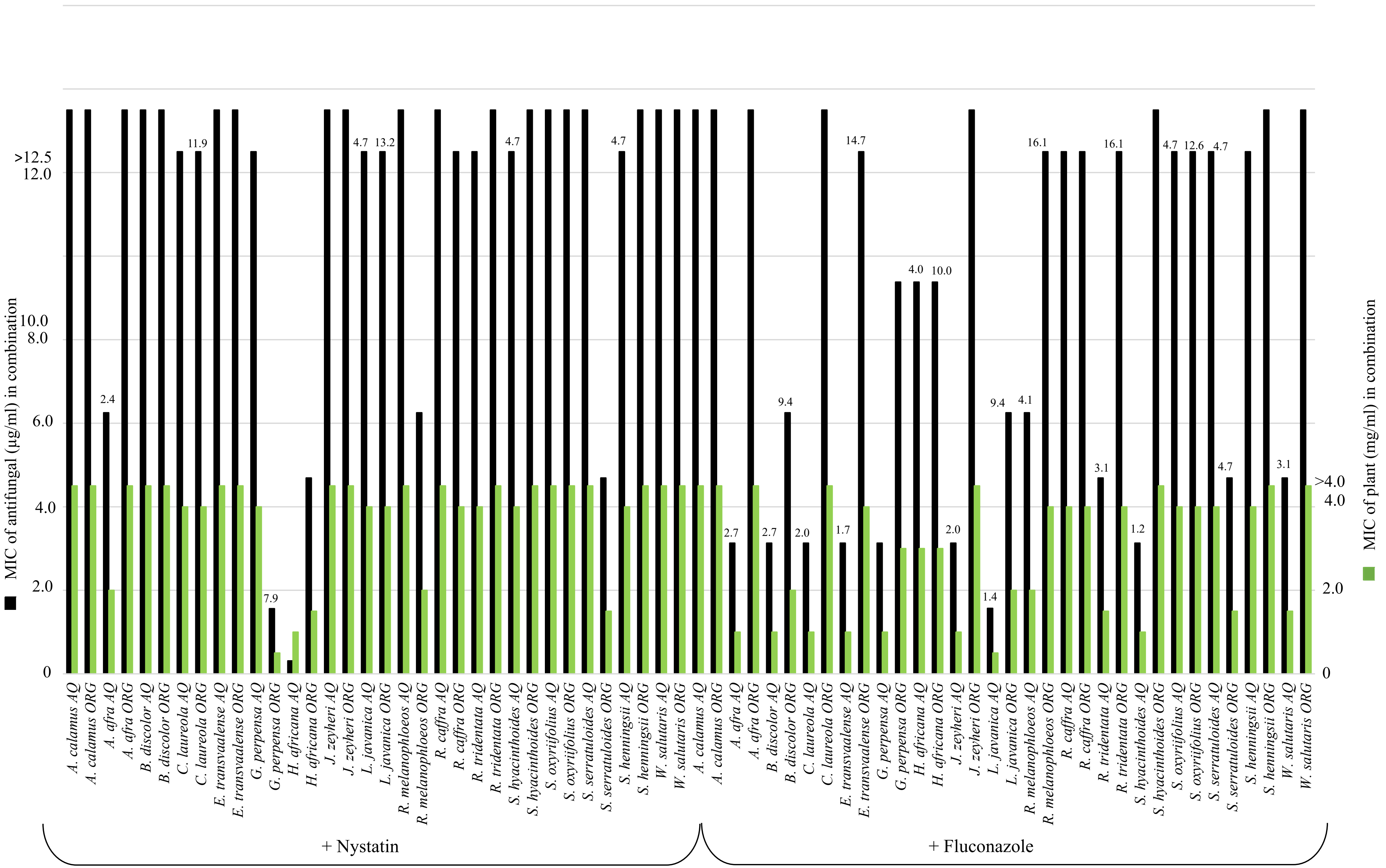

Figure S19. Plant extracts and conventional antifungals against *Candida glabrata.*

Individual MIC values in tables as mg/ml. Values indicated above columns ΣFIC could be determined. No value indicated above column ΣFIC could not be determined.

(*AQ*) aqueous extract. (*ORG*) organic extract. Acetone in water (32.0 mg/ml) as the negative control (MIC >8.0 mg/ml). Nystatin (0.1 mg/ml) as the positive control (MIC 5.210 µg/ml). Culture in TSB as the culture control (MIC >8.0 mg/ml).
